# Integrative pathway and network analysis provide insights on flooding-tolerance genes in soybean

**DOI:** 10.1038/s41598-023-28593-1

**Published:** 2023-02-03

**Authors:** Li-Hsin Jhan, Chin-Ying Yang, Chih-Min Huang, Mu-Chien Lai, Yen-Hsiang Huang, Supaporn Baiya, Chung-Feng Kao

**Affiliations:** 1 Department of Agronomy, College of Agriculture and Natural Resources, National Chung Hsing University, Taichung, Taiwan; 2 Physiology and Biochemistry Division, Taiwan Banana Research Institute, Pingtung, Taiwan; 3 Department of Resource and Environment Faculty of Science at Sriracha, Kasetsart University at Sriracha Campus, Sriracha, 20230 Chonburi Thailand; 4 Advanced Plant Biotechnology Center, National Chung Hsing University, Taichung, Taiwan

**Keywords:** Plant sciences, Systems biology

## Abstract

Soybean is highly sensitive to flooding and extreme rainfall. The phenotypic variation of flooding tolerance is a complex quantitative trait controlled by many genes and their interaction with environmental factors. We previously constructed a gene-pool relevant to soybean flooding-tolerant responses from integrated multiple omics and non-omics databases, and selected 144 prioritized flooding tolerance genes (FTgenes). In this study, we proposed a comprehensive framework at the systems level, using competitive (hypergeometric test) and self-contained (sum-statistic, sum-square-statistic) pathway-based approaches to identify biologically enriched pathways through evaluating the joint effects of the FTgenes within annotated pathways. These FTgenes were significantly enriched in 36 pathways in the Gene Ontology database. These pathways were related to plant hormones, defense-related, primary metabolic process, and system development pathways, which plays key roles in soybean flooding-induced responses. We further identified nine key FTgenes from important subnetworks extracted from several gene networks of enriched pathways. The nine key FTgenes were significantly expressed in soybean root under flooding stress in a qRT-PCR analysis. We demonstrated that this systems biology framework is promising to uncover important key genes underlying the molecular mechanisms of flooding-tolerant responses in soybean. This result supplied a good foundation for gene function analysis in further work.

## Introduction

Soybean [*Glycine max* (L.) Merr] provides abundant flavonoids, plant-based proteins and lipids. It is the major protein source for vegetarians. Soybean is nutritious for their isoflavones and anthocyanins belonging to flavonoid compounds^1^. Isoflavones, of which soybean has higher content, generally exist in many kinds of plants^2^. Isoflavones have been functionally linked to anti-oxidation, reduction in inflammation, inhibition of free radicals, and cancer prevention^3–5^. Anthocyanin and its main constituents, such as cyaniding-3-*O*-glucoside, present in soybeans can effectively inhibit lipopolysaccharide, hydrogen peroxide, and pro-inflammatory cytokines, which are a natural source of antioxidants and anti-inflammatory^6–8^. Hence, soybeans could be used to boost the nutritional content, nutraceutical products, and potential therapeutic agents for some pathological diseases.

Soybeans are highly sensitive to growth conditions, particularly in flooding environments^9,10^. In recent years, global agriculture damage and losses from changing climate (e.g. flooding) have increased^11,12^. Extreme torrential rain or momentary heavy rain brought by strong southwesterly air currents or jet streams induced by typhoons has caused severe flooding during soybean (including edamame) autumn seedlings in southern and western areas of Taiwan. In the United States, flooding can occur sequentially during a single crop cycle or independently in the same fields during different years. Over the past 15 years, flooding resulted in $6.2 billion worth of soybean production losses. Loss of soil, nutrients, and pesticides to waterways is a major problem in high agricultural production areas such as the Mid-western United States^13,14^. In China, the flooding stress of soybean is associated with excess irrigation that impairs water uptake, and soil waterlogging is largely affected by the season^15^. The total summer crop sown area in 2020 is 26.17 million hectares; therefore, the floods affected 23% of the planted area of summer crops and caused 4.3% of crop failure. Facing such high uncertainty climate change, we need a systematical and comprehensive method to find the whole picture of defense mechanisms against flooding for breeding stress-tolerant cultivars.

There is general recognition that flooding can be classified into waterlogging, when the water covers only the root system, and submergence, when the water covers both the shoot and the root system, according to water levels above the soil surface^16^. The present study mainly focuses on submergence. Abiotic stresses can disturb plant growth and adversely affect growth characteristics, for example, leaf etiolation and the number of pods per plant^17–19^. Under the flooding stress, the contents of flavonoid compounds in soybean increase significantly, but the yields decrease simultaneously^20–22^. Also, cell wall maturation, cell wall formation, and plant development will be seriously changed during flooding^23–26^. Thus, a better understanding of the physiological mechanisms involved in flooding-induced response and tolerance of soybeans is needed for breeding work.

Mechanisms related to flooding tolerance or response have been investigated and reviewed^27,28^. At initial flooding stress of soybean, ATP-citrate lyase and xylosidase decrease while alcohol dehydrogenases and calreticulin increase^29^. These enzymes are related to the tricarboxylic acid cycle, cell wall maturation, alcohol fermentation, and calcium homeostasis^26,30,31^. Prolonged submergence caused a significant decline in photosynthesis, stomatal conductance, and the nutrition absorption of leaves^32^. Soybean produces abscisic acid (ABA) to regulate protein kinases under hypoxia^33^. These protein kinases are related to pathways including glycolysis, cell organization, and vesicle transport^20,22,33^. The proteomic analyses have found that excessive water supply for soybean roots induces anthocyanin 5-aromatic acyltransferase, anthocyanin malonyltransferase, and isoflavone reductase to increase^20,34,35^. These protein kinases facilitate isoflavones and anthocyanins to increase the survival rate after flooding. Although many molecular and physiological mechanisms were reported, mechanisms of flooding-induced response and tolerance have yet to be fully clarified for soybean. No studies were reported on the enhancement of pathway analysis for flooding tolerance and response, a polygenetic trait, by introducing multigenes selected from an integrated knowledge framework in a systematic and comprehensive design^36^.

Flooding tolerance is a complex quantitative (or polygenic) trait, which is regulated through several biological pathways that are controlled by a number of genes (i.e., polygenes). Many functional mechanisms studies for flooding tolerance in soybean have been reported^20,33,37^. Most of the studies were based on selected candidate genes that were hypothesis-driven, such as text-mining-based^38^ and meta-analysis-based^39^. However, these mechanisms may only partially explain flooding due to a limited understanding of the genetic make-up of a polygenic trait, particularly flooding tolerance. Furthermore, potential biases might have affected the results using the hypothesis-free approach, for example, genome-wide association study (GWAS), because it is challenging to account for variations between germplasms and quantitative trait^40^. It is also challenging to balance the results between false positives and false negatives in GWAS^41^. Determining the genetic makeup underlying flooding tolerance in soybean is crucial to precisely identifying mechanisms related to flooding tolerance or responding to stress. Hence, applying pathway-based analysis to selected candidate genes can systematically integrate prior biological knowledge of gene regulating functions and biological pathway information or functional categories to figure out the whole picture of physiological mechanisms for flooding tolerance in soybean. This can reveal a more comprehensive picture at the molecular level than a single marker-based or gene-level analysis.

The main purpose of system biology is to precisely explore the unknown mechanisms in experimental data containing implicit biological information^42^. Through systematical methods, pathway enrichment analysis, and network analysis, for example, enable us to understand the signal transmission of responses biologically in a plant cell being stimulated by an environmental factor. These signals are complicated, information-worthless in a single signal but information-valuable in systematic manners^43^. Pathway enrichment analysis, a knowledge-based approach, provide biological insights into molecular responses to a trait of interest from integrated omics and non-omics (OnO) data^44^. Pathway enrichment analysis detects whether particular biological pathways or molecular groups are significantly overrepresented. Networks have successfully carried on the idea of graph theory and probability theory to succinctly represent a mathematical structure of biological components using a group of nodes (e.g. proteins, genes, pathways) and links (e.g. genetic and/or functional interactions)^45^. Using available biological knowledge and candidate genes selected from integrated OnO data for network analysis provides a great potential to uncover novel information on complex biological networks^46^.

Methods of pathway enrichment analysis in systems biology can be generalized into, but not limited to, competitive and self-contained method^47^. In the competitive method, it compares associations between two gene sets (i.e., genes in a specific pathway versus genes not in that pathway) and traits, such as a hypergeometric test^48^. However, the self-contained method only considers associations between the genes in a specific pathway and traits, such as sum-statistic (e.g. SUMSTAT) and sum-square (e.g. SUMSQ) statistic^49^. There are several examples that successfully applied pathway-based analysis to explore potential mechanisms and biological functions for important traits in plants, including cytoplasmic male sterile in soybean^50^, comparison between a mutant gene and wild-type in soybean^51^, or high temperature in soybean^52^. Recently, Naithani et al.^53^ developed the Plant Reactome, a knowledgebase and resource for pathway-based analysis in plants to address important biological questions and regulatory mechanisms. Many open-access knowledgebase data such as Gene Ontology (GO, http://geneontology.org/) and Kyoto Encyclopedia of Genes Genomes (KEGG, https://www.genome.jp/kegg/kegg2.html) are commonly used worldwide. These functional annotations provide opportunities to access the whole map underlying a specific trait via systematically testing unknown functional gene sets by statistical model^54^. The networks integrate biological information (e.g. proteins, molecules, pathways), and quantify nucleic acid information, providing information on the associations between several genetic loci, and how genes and pathways interact with each other (i.e., gene modules) to regulate traits. The association between genes can be visualized by the network composed of nodes and edges, making the complex associations between genes presented in a simple and trivial way^55,56^. It is practical and efficient way to reveal enriched pathways and networks for flooding-tolerant responses using candidate genes prioritized from integrated OnO databases^57^.

**Figure 1:**
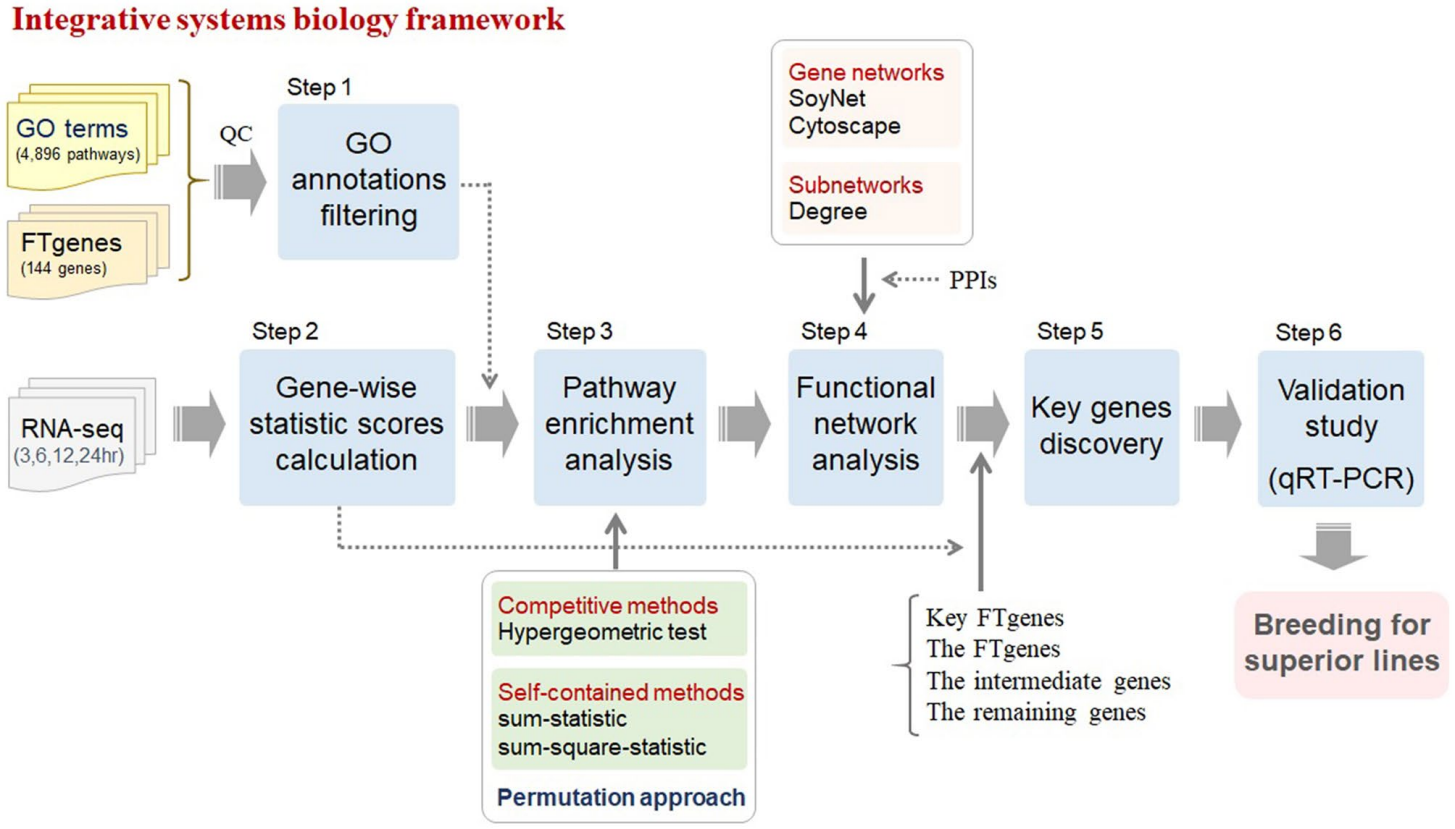
The study pipelines. This pipeline consists of six steps. The first step is the GO annotations filtering. The second step is gene-wise statistic score calculation. The third step is the pathway enrichment analysis. The fourth step is the gene network construction. The fifth step is the discovery of key genes. The final step is the validation study using the qRT-PCR experiments.

**Figure 2:**
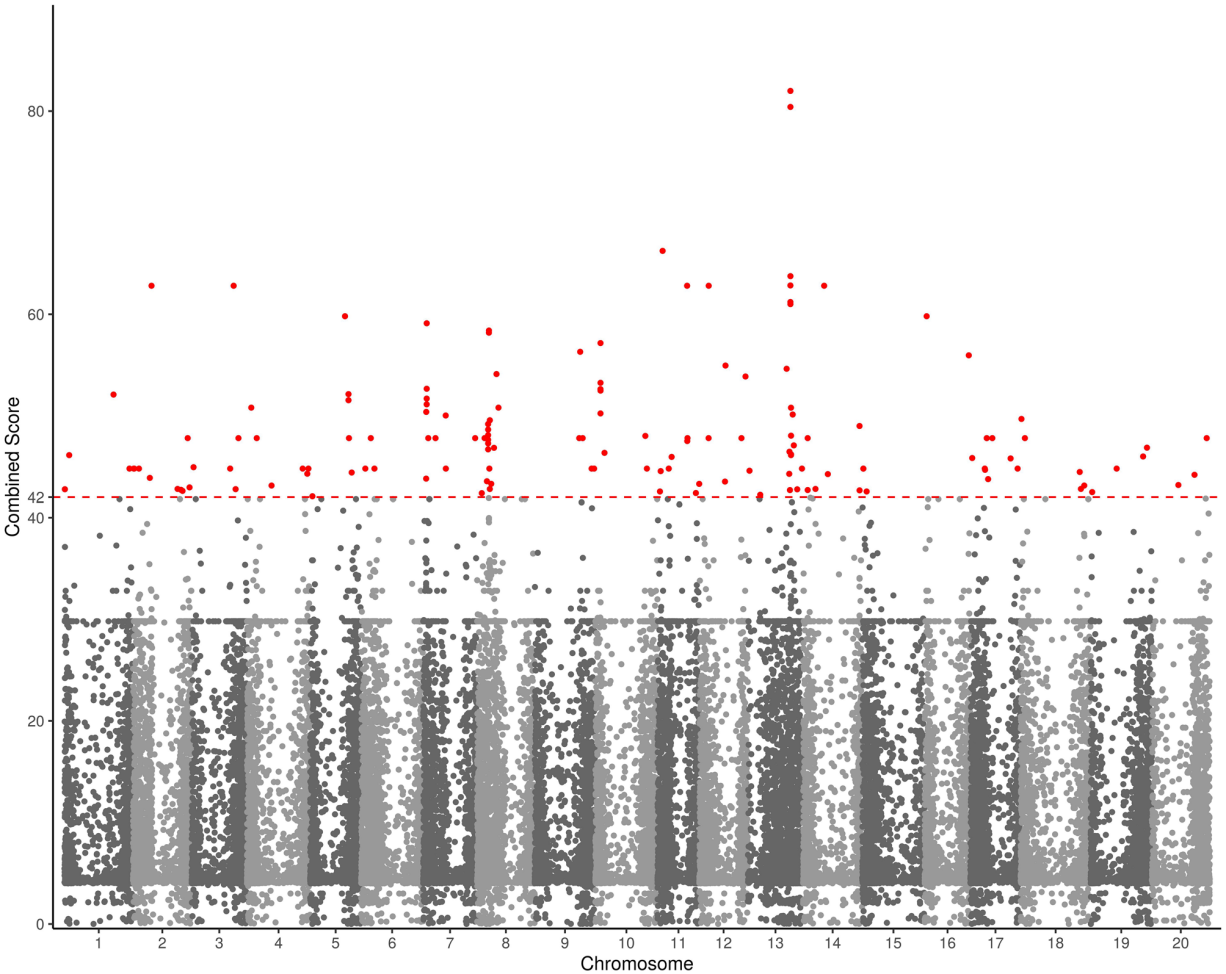
Manhattan plot of the flooding-tolerance genes in soybean. The dashed line is the cut-off score of 42. Dots colored in red are the FTgenes.

We previously developed a comprehensive framework to integrate OnO data that is relevant to flood-tolerant responses in soybean. A total of 36,705 genes were collected and prioritized according to their magnitude of association with flooding-tolerant responses^36^. In this study, we introduced a systems biology framework (Fig. 1), through the pathway enrichment analysis (both the competitive and the self-contained methods) and network analysis to combine the joint effects of the 144 prioritized flooding tolerance genes (i.e., FTgenes) (Fig. 2) to uncover the molecular mechanisms underlying flooding-tolerant responses in soybean. The strategies proposed in this study can better understanding in how flooding-tolerance genes act against a flooding event and protect soybean plants from floods in complex biological systems.

## Results

### Gene-pathway mapping

A total of 14,772, 17,017, 19,060, and 18,889 expression data (Step 1 in Fig. 1) from soybean roots after 3, 6, 12, and 24 h (h) of submergence treatments in the RNA-seq database^58^ were used as the test sets to conduct pathway enrichment analyses. Only pathways (i.e. GO terms) containing at least one FTgenes (Fig. 2) were considered, resulting in 417 annotated pathways (Step 1 in Fig. 1) for pathway enrichment analysis.

### Gene-wise statistic values

**Figure 3:**
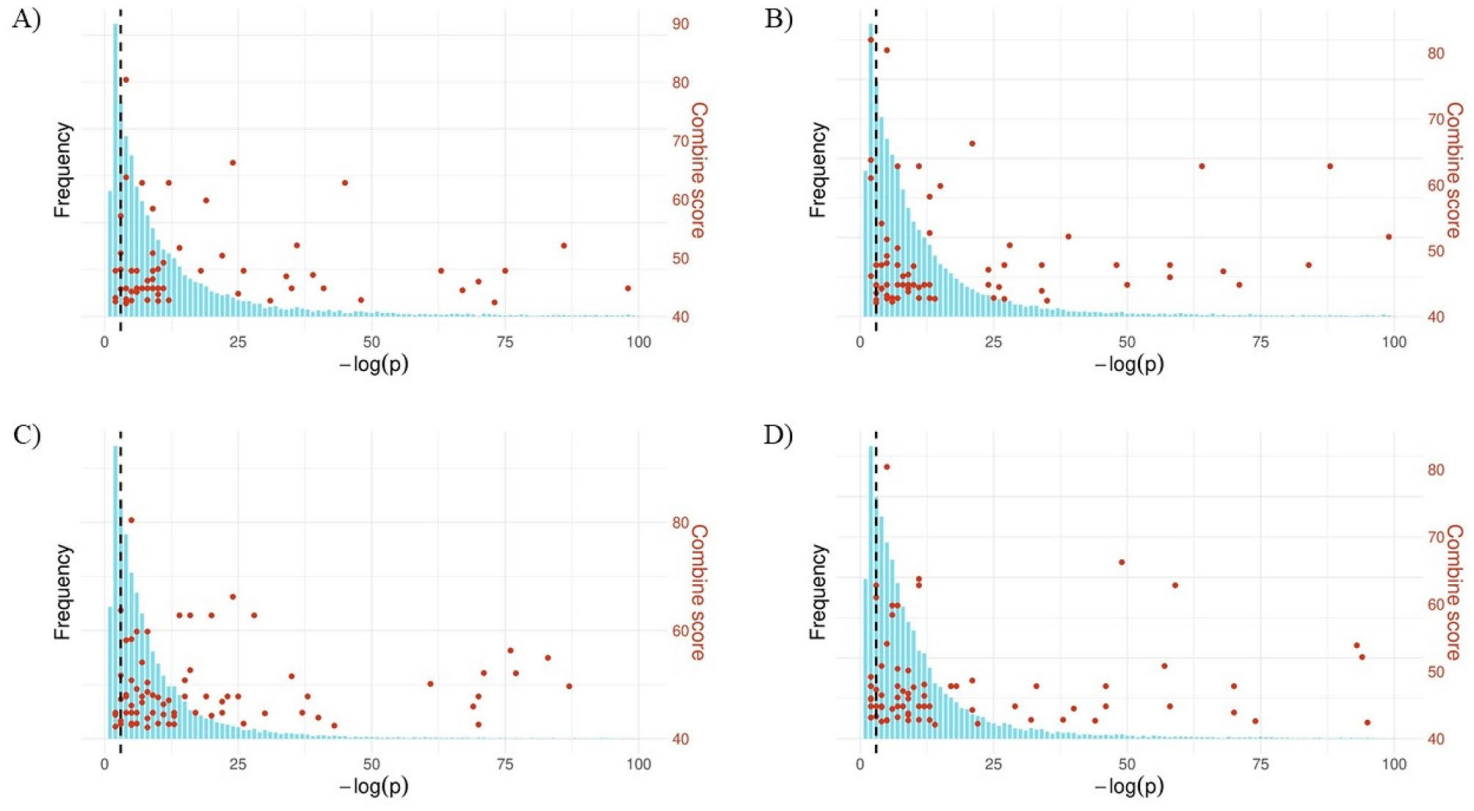
Expression skewness of gene-wise statistic scores. The p-values were transformed into 10-based logarithms for capturing changes in expressions of soybean roots flooded after (**A**) 3 h, (**B**) 6 h, (**C**) 12 h, and (**D**) 24 h. The red solid dots represent the FTgenes with a combined score greater than 42. The dashed line represents − log(p-value) = 3.

For gene score calculation, we transformed expression-level statistics (i.e. p-values) using 10-based logarithms into gene-wise statistic scores (Step 2 in Fig. 1) to measure changes in gene expression in roots flooded after 3, 6, 12, and 24 h. The distributions of gene-wise statistic scores were highly skewed to the right (Fig. 3), as seen in the microarray data. The expression skewness of each dataset was 4.82, 4.31, 4.91, and 4.24, respectively, indicating the expression skewness has the potential to reveal new insights into the FTgenes (Fig. 2) in the analyses of pathway enrichment and gene network.

### Competitive method (hypergeometric test) revealed the mechanisms of flooding-tolerant responses

**Figure 4:**
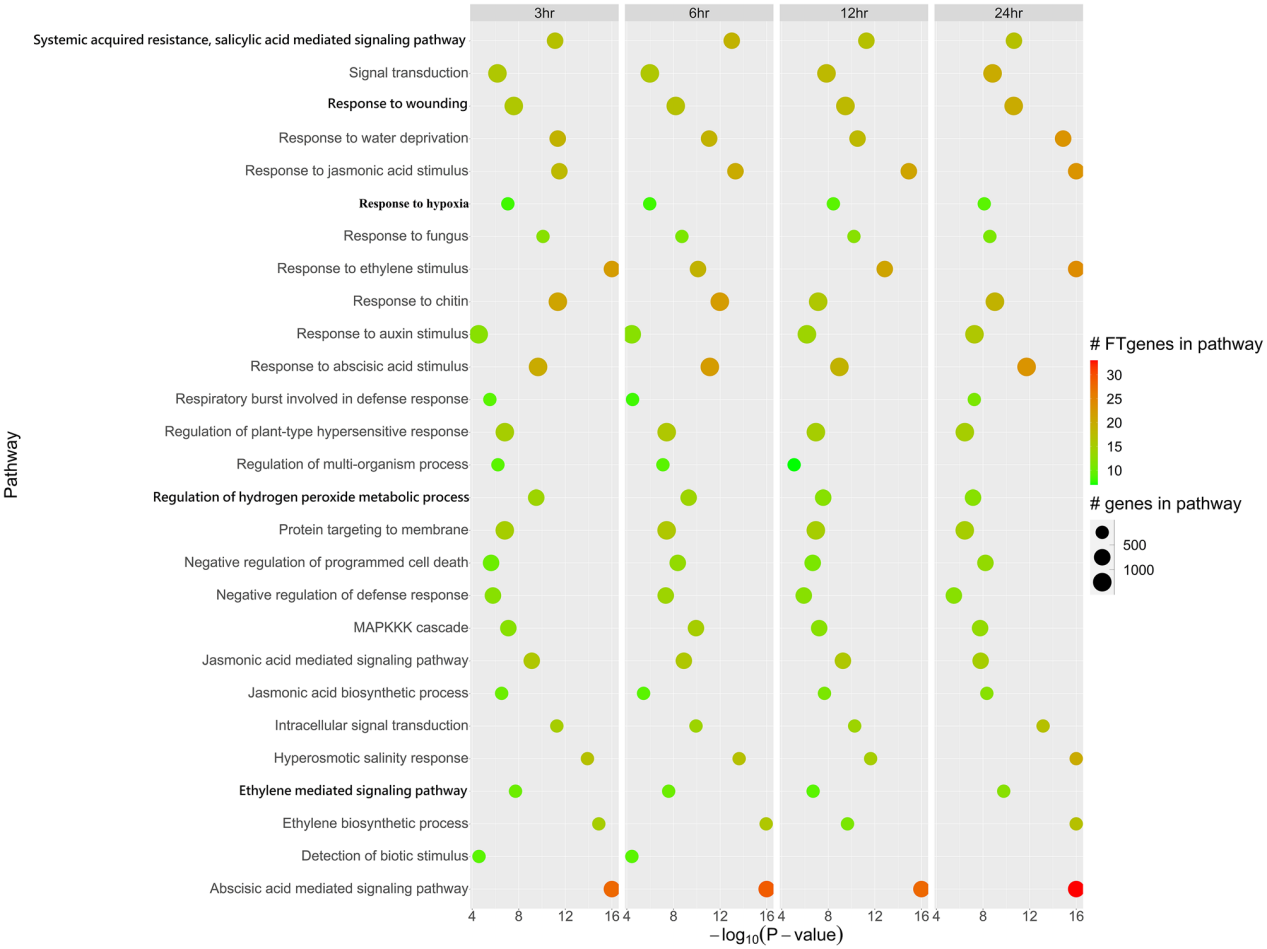
Pathway enrichment analysis of the FTgenes using the competitive method. Enriched GO pathways were identified by using the hypergeometric test. Software R v3.6.2 (https://www.r-project.org/) was used to create image.

Using the hypergeometric model test (Step 3 in Fig. 1), we initially found 27 pathways (Fig. 4) with at least one nominal p-value less than 1.00 × 10^–4^ that were enriched with flooding tolerance or response to the stress in the gene expression data from soybean roots after 3, 6, 12, and 24 h of submergence treatments. Among them, 24 pathways were significantly enriched at all four-time points after submergence treatments. Table 1 demonstrated detailed information on significantly enriched pathways overrepresented in the gene expression dataset. The top five pathways included ‘abscisic acid mediated signaling pathway’, ‘response to ethylene stimulus’, ‘ethylene biosynthetic process’, ‘hyperosmotic salinity response’, and ‘response to the jasmonic acid stimulus’. Two pathways, ‘abscisic acid mediated signaling pathway’ and ‘response to ethylene stimulus’, were the most significantly enriched at 3 h after submergence treatments. The pathway of ‘abscisic acid mediated signaling pathway’ was the most significantly enriched at 6 and 12 h after submergence treatments. The top five pathways were the most significantly enriched at 24 h after submergence treatments.

**Table 1:** Significantly enriched pathways in gene expression data for flooding-tolerance using hypergeometric test.

Annotated pathway	N_G_	3 h		6 h		12 h		24 h	
		N_FT_	p-value^a^	N_FT_	p-value^a^	N_FT_	p-value^a^	N_FT_	p-value^a^
Abscisic acid mediated signaling pathway	630	28	** < 1.00 × 10**^**–16**^	29	** < 1.00 × 10**^**–16**^	28	** < 1.00 × 10**^**–16**^	33	** < 1.00 × 10**^**–16**^
Response to ethylene stimulus	723	22	** < 1.00 × 10**^**–16**^	19	**7.86 × 10**^**–11**^	21	**1.42 × 10**^**–13**^	24	** < 1.00 × 10**^**–16**^
Ethylene biosynthetic process	268	15	**1.33 × 10**^**–15**^	16	**1.11 × 10**^**–16**^	11	**2.22 × 10**^**–10**^	17	** < 1.00 × 10**^**–16**^
Hyperosmotic salinity response	459	17	**1.25 × 10**^**–14**^	17	**2.31 × 10**^**–14**^	15	**2.32 × 10**^**–12**^	20	** < 1.00 × 10**^**–16**^
Response to jasmonic acid stimulus	754	18	**3.25 × 10**^**–12**^	20	**4.80 × 10**^**–14**^	21	**1.22 × 10**^**–15**^	23	** < 1.00 × 10**^**–16**^
Response to water deprivation	883	19	**4.50 × 10**^**–12**^	19	**8.70 × 10**^**–12**^	18	**3.06 × 10**^**–11**^	23	**1.33 × 10**^**–15**^
Response to chitin	1130	21	**4.41 × 10**^**–12**^	22	**1.06 × 10**^**–12**^	16	**7.46 × 10**^**–8**^	19	**9.72 × 10**^**–10**^
Intracellular signal transduction	476	15	**5.37 × 10**^**–12**^	14	**1.17 × 10**^**–10**^	14	**5.40 × 10**^**–11**^	17	**7.02 × 10**^**–14**^
Systemic acquired resistance, salicylic acid mediated signaling pathway	684	17	**7.53 × 10**^**–12**^	19	**9.99 × 10**^**–14**^	17	**5.34 × 10**^**–12**^	17	**2.23 × 10**^**–11**^
Response to fungus	310	12	**8.21 × 10**^**–11**^	11	**1.90 × 10**^**–9**^	12	**6.25 × 10**^**–11**^	11	**2.62 × 10**^**–9**^
Response to abscisic acid stimulus	1249	20	**2.20 × 10**^**–10**^	22	**7.58 × 10**^**–12**^	19	**1.11 × 10**^**–9**^	23	**1.84 × 10**^**–12**^
Regulation of hydrogen peroxide metabolic process	532	14	**3.13 × 10**^**–10**^	14	**4.97 × 10**^**–10**^	12	**2.73 × 10**^**–8**^	12	**7.24 × 10**^**–8**^
Jasmonic acid mediated signaling pathway	797	16	**7.62 × 10**^**–10**^	16	**1.29 × 10**^**–9**^	16	**5.51 × 10**^**–10**^	15	**1.62 × 10**^**–8**^
Ethylene mediated signaling pathway	309	10	**1.88 × 10**^**–8**^	10	**2.59 × 10**^**–8**^	9	**2.00 × 10**^**–7**^	12	**1.68 × 10**^**–10**^
Response to wounding	1025	16	**2.67 × 10**^**–8**^	17	**6.43 × 10**^**–9**^	18	**3.40 × 10**^**–10**^	20	**2.37 × 10**^**–11**^
MAPKKK cascade	572	12	**7.82 × 10**^**–8**^	15	**1.17 × 10**^**–10**^	12	**6.02 × 10**^**–8**^	13	**1.80 × 10**^**–8**^
Response to hypoxia	194	8	**8.54 × 10**^**–8**^	8	**1.10 × 10**^**–7**^	9	**3.66 × 10**^**–9**^	9	**7.87 × 10**^**–9**^
Regulation of plant-type hypersensitive response	1015	15	**1.57 × 10**^**–7**^	16	**3.86 × 10**^**–8**^	15	**1.16 × 10**^**–7**^	15	**3.70 × 10**^**–7**^
Protein targeting to membrane	1016	15	**1.59 × 10**^**–7**^	16	**3.91 × 10**^**–8**^	15	**1.18 × 10**^**–7**^	15	**3.74 × 10**^**–7**^
Negative regulation of defense response	762	12	**1.63 × 10**^**–6**^	14	**4.68 × 10**^**–8**^	12	**1.27 × 10**^**–6**^	12	**3.19 × 10**^**–6**^
Signal transduction	1301	16	**6.76 × 10**^**–7**^	16	**1.08 × 10**^**–6**^	18	**1.43 × 10**^**–8**^	20	**1.53 × 10**^**–9**^
Response to auxin stimulus	1009	12	**2.77 × 10**^**–5**^	12	**3.86 × 10**^**–5**^	14	**6.87 × 10**^**–7**^	16	**5.50 × 10**^**–8**^
Jasmonic acid biosynthetic process	415	10	**2.94 × 10**^**–7**^	9	**3.73 × 10**^**–6**^	11	**2.12 × 10**^**–8**^	12	**4.72 × 10**^**–9**^
Negative regulation of programmed cell death	523	10	**2.37 × 10**^**–6**^	13	**4.33 × 10**^**–9**^	11	**2.18 × 10**^**–7**^	13	**6.26 × 10**^**–9**^
Respiratory burst involved in defense response	418	9	**3.01 × 10**^**–6**^	8	**3.25 × 10**^**–5**^	7	1.64 × 10^–4^	11	**5.64 × 10**^**–8**^
Regulation of multi-organism process	262	9	**6.10 × 10**^**–8**^	9	**8.11 × 10**^**–8**^	7	**8.56 × 10**^**–6**^	6	1.47 × 10^–4^
Detection of biotic stimulus	276	9	**2.56 × 10**^**–5**^	9	**3.72 × 10**^**–5**^	7	3.80 × 10^–3^	6	6.16 × 10^–2^

*N*_*G*_ total number of genes in a specific pathway (i.e. GO term), *N*_*FT*_ number of overlap genes between FTgenes and pathway.

^a^Pathways with p-values, calculated using competitive method (hypergeometric test), less than 1.00 × 10^–4^ were significantly enriched with flooding-tolerant responses in soybean.

Significant values are in bold.

### Self-contained methods (SUMSTAT, SUMSQ) revealed the mechanisms of flooding-tolerant responses

**Figure 5:**
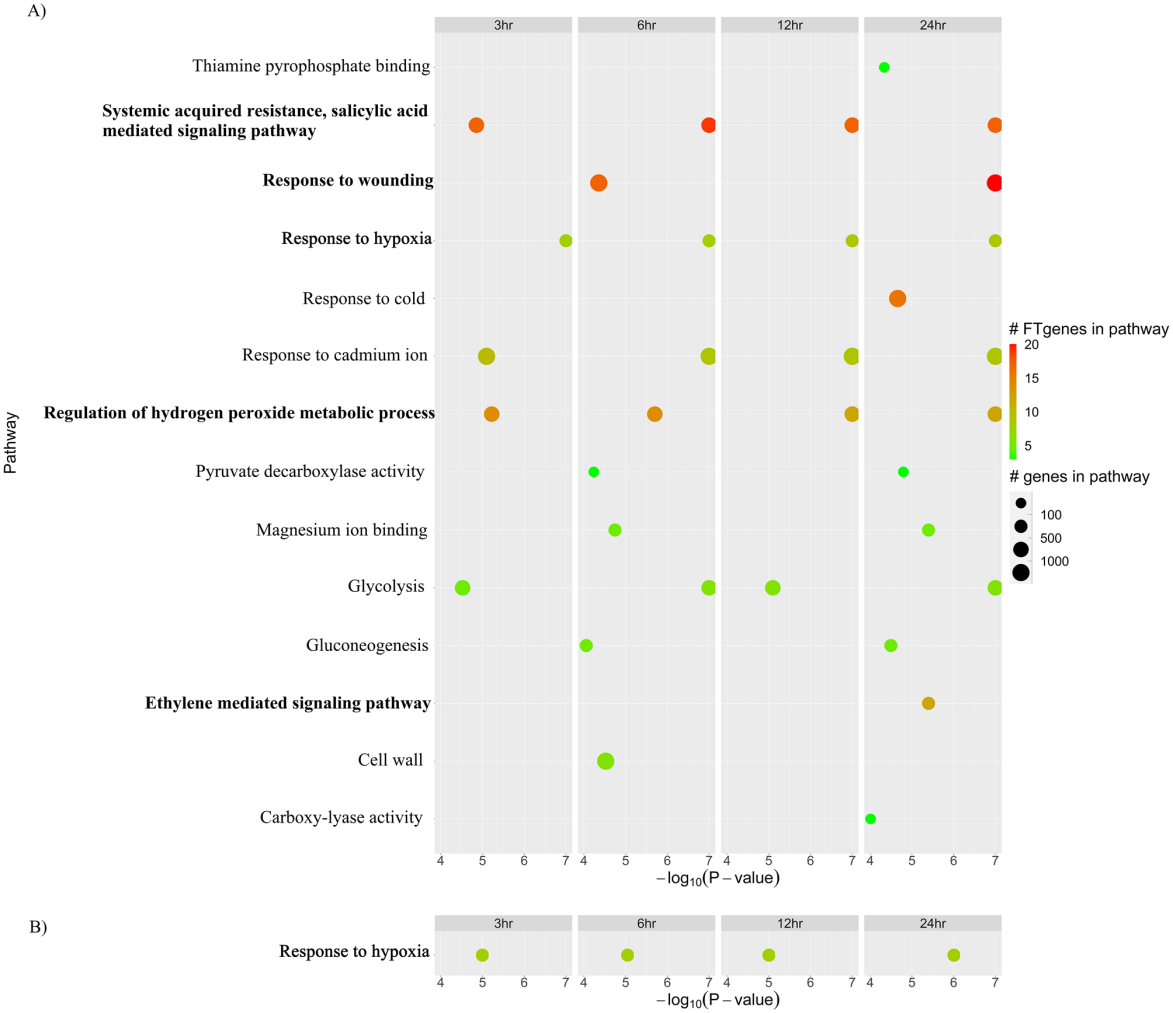
Pathway enrichment analysis of the FTgenes using the self-contained methods. Enriched GO pathways were identified based on 10,000 permutations by using (**A**) SUMSTAT and (**B**) SUMSQ statistics. Software R v3.6.2 (https://www.r-project.org/) was used to create image.

The 144 FTgenes (Fig. 2) were significantly enriched in fourteen GO pathways (14 in SUMSTAT and 1 in SUMSQ) after controlling the false discovery rate at the 0.05 level in the self-contained approaches (Step 3 in Figs. 1, 5). Among them, only one GO pathway, ‘response to hypoxia’, was found at all four-time points in both methods. Tables 2 and 3 demonstrated detailed information of significantly enriched pathways overrepresented in the gene expression dataset using SUMSTAT and SUMSQ, respectively. Five GO pathways were significantly enriched after submergence treatments at all four-time points. The top five pathways included ‘response to hypoxia’, ‘response to cadmium ion’, ‘systemic acquired resistance’, ‘salicylic acid mediated signaling pathway’, ‘regulation of hydrogen peroxide metabolic process’, and ‘glycolysis’. One pathway, ‘response to hypoxia’, was the most significantly enriched at 3 h after submergence treatments. Three pathways, including ‘response to hypoxia’, ‘systemic acquired resistance, salicylic acid mediated signaling pathway’, and ‘response to cadmium ion’ were the most significantly enriched at both 6 and 12 h after submergence treatments. The ‘response to wounding’ pathway and the top five pathways were the most significantly enriched at 24 h after submergence treatments. Six pathways (‘response to wounding’, ‘ethylene mediated signaling pathway’, ‘carboxy-lyase activity’, ‘thiamine pyrophosphate binding’, ‘response to cold’, and ‘cell wall’) were not enriched at 3 h at the beginning but enriched later during 6–24 h after submergence treatments.

**Table 2:** Significantly enriched pathways in gene expression data for flooding-tolerance using SUMSTAT statistic.

Pathway	N_G_	3 h		6 h		12 h		24 h	
		N_FT_	p-value^a^	N_FT_	p-value^a^	N_FT_	p-value^a^	N_FT_	p-value^a^
Response to hypoxia	194	8	** < 1.00 × 10**^**–7**^	8	** < 1.00 × 10**^**–7**^	9	** < 1.00 × 10**^**–7**^	9	** < 1.00 × 10**^**–7**^
Response to cadmium ion	1301	10	**8.00 × 10**^**–6**^	9	** < 1.00 × 10**^**–7**^	9	** < 1.00 × 10**^**–7**^	9	** < 1.00 × 10**^**–7**^
Systemic acquired resistance, salicylic acid mediated signaling pathway	684	17	**1.40 × 10**^**–5**^	19	** < 1.00 × 10**^**–7**^	17	** < 1.00 × 10**^**–7**^	17	** < 1.00 × 10**^**–7**^
Regulation of hydrogen peroxide metabolic process	532	14	**6.00 × 10**^**–6**^	14	**2.00 × 10**^**–6**^	12	** < 1.00 × 10**^**–7**^	12	** < 1.00 × 10**^**–7**^
Glycolysis	658	5	**3.00 × 10**^**–5**^	6	** < 1.00 × 10**^**–7**^	6	**8.00 × 10**^**–6**^	6	** < 1.00 × 10**^**–7**^
Response to wounding	1025	16	1.00	17	**4.40 × 10**^**–5**^	18	2.84 × 10^–3^	20	** < 1.00 × 10**^**–7**^
Magnesium ion binding	247	5	1.04 × 10^–4^	5	**1.80 × 10**^**–5**^	5	1.52 × 10^–4^	5	**4.00 × 10**^**–6**^
Gluconeogenesis	462	5	2.60 × 10^–4^	5	**8.80 × 10**^**–5**^	5	8.56 × 10^–4^	5	**3.20 × 10**^**–5**^
Pyruvate decarboxylase activity	18	3	1.22 × 10^–3^	3	**5.80 × 10**^**–5**^	3	1.20 × 10^–4^	3	**1.60 × 10**^**–5**^
Ethylene mediated signaling pathway	309	10	1.00	10	2.00 × 10^–4^	9	1.76 × 10^–4^	12	**4.00 × 10**^**–6**^
Carboxy-lyase activity	38	3	1.00	3	3.32 × 10^–4^	3	4.08 × 10^–4^	3	**9.80 × 10**^**–5**^
Thiamine pyrophosphate binding	29	3	1.00	3	1.04 × 10^–4^	3	4.34 × 10^–4^	3	**4.60 × 10**^**–5**^
Response to cold	1113	11	1.00	13	1.65 × 10^–3^	15	2.92 × 10^–3^	16	**2.20 × 10**^**–5**^
Cell wall	1355	6	1.00	6	**3.00 × 10**^**–5**^	6	1.26 × 10^–4^	5	1.49 × 10^–3^

*N*_*G*_ total number of genes in a specific pathway (i.e. GO term), *N*_*FT*_ number of overlap genes between FTgenes and pathway.

^a^Pathways with p-values, calculated based on 10,000 permutations using self-contained method (SUMSTAT statistic), less than 1.00 × 10^–4^ were significantly enriched with flooding-tolerant responses in soybean.

Significant values are in bold.

**Table 3:** Significantly enriched pathways in gene expression data for flooding-tolerance using SUMSQ statistic.

Pathway	N_G_	3 h		6 h		12 h		24 h	
		N_FT_	p-value^a^	N_FT_	p-value^a^	N_FT_	p-value^a^	N_FT_	p-value^a^
Response to hypoxia	194	8	6.00 × 10^–5^	8	4.20 × 10^–5^	9	1.00 × 10^–5^	9	< 1.00 × 10^–6^

*N*_*G*_ total number of genes in a specific pathway (i.e. GO term), *N*_*FT*_ number of overlap genes between FTgenes and pathway.

^a^Pathways with p-values, calculated based on 10,000 permutations using self-contained method (SUMSQ statistic), less than 1.00 × 10^–4^ were significantly enriched with flooding-tolerant responses in soybean.

We found that four pathways (response to hypoxia, systemic acquired resistance, salicylic acid mediated signaling pathway, regulation of hydrogen peroxide metabolic process, and response to wounding) were reported in both competitive and self-contained approaches. However, there was no overlap among the top five pathways in both approaches.

### Gene network analysis selects the key genes relevant to flooding-tolerant responses

**Figure 6:**
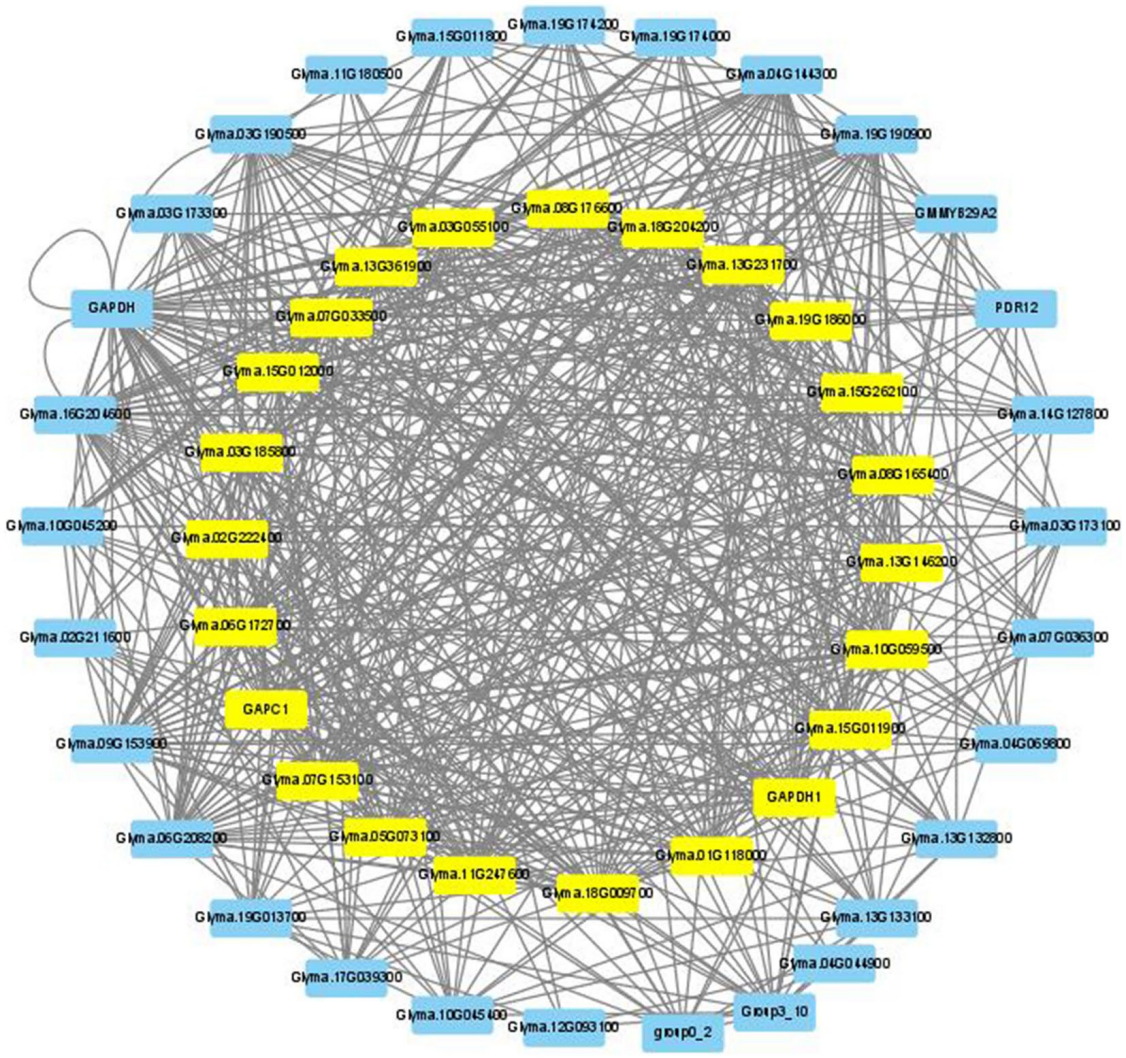
The gene network analysis of the FTgenes. 103 FTgenes were selected from enriched pathways to construct the gene network. Software Cytoscape v3.9.0 (https://cytoscape.org/) was used to create image.

Since many FTgenes were involved in flooding-tolerant responses, we conducted a functional gene network analysis (Step 4 in Fig. 1) to better understand how these FTgenes work together. Among the 144 FTgenes, 103 were found to have protein–protein interactions (PPIs) in the soybean interactome. Using the functional modules analytic tool in SoyNet, we successfully constructed a gene network specific to flooding-tolerant responses in soybean (Fig. 6; Sheet 1 in Supplementary Table 1). This gene network contained 103 FTgenes and 70 intermediate genes that were highly connected nodes (hubs) in the reference network and hence recruited in the gene network. The degree values of the 173 genes ranged from 0 to 66, with an average degree of 7.32. Of which, 110 genes (degree values between 0 and 2) and 13 genes (degree values between 3 and 10) had a low degree of centrality and were hence excluded from the gene network. Figure 6 demonstrated a dense gene network containing 50 genes, of which 23 genes had degree values between 20 and 30, demonstrating a high degree of centrality in the gene network. The 23 genes (highlighted in yellow), including eight FTgenes and 15 significant intermediate genes, had an average degree of 25.5. Among them, the eight FTgenes (*Glyma.02g222400*, *Glyma.18g009700*, *Glyma.13g231700*, *Glyma.13g361900*, *Glyma.15g012000*, *Glyma.07g153100*, *Glyma.01g118000*, and *Glyma.15g011900*), reported in both competitive and self-contained pathway analytic strategies (Table 4), demonstrated a high degree of centrality ranged between 20 and 30 (the average degree was 26). The eight FTgenes are mainly related to signal transduction (*Glyma.13g361900*, *Glyma.15g011900*, and *Glyma.15g012000*), energy-producing (*Glyma.02g222400*, *Glyma.13g361900*, and *Glyma.18g009700*), and plant hormone regulation (*Glyma.15g011900* and *Glyma.15g012000*), indicating they play important roles in flooding-tolerant responses in soybean.

**Table 4:** Contributions of the FTgenes in three pathway analysis.

Gene	N^pw^	N^method^	Gene size (kb)	Z-score^a^	Annotation	Source
*Glyma.02g195300*	23	3	2.723	10.62	Intracellular protein transport	Uniprot GO
*Glyma.05g021100*	20	3	6.774	0		
*Glyma.08g218600*	20	3	2.408	19.59	Regulation of defense response	Arabidopsis GO
*Glyma.14g102900*	20	3	2.732	0		
*Glyma.13g234500*	19	3	1.059	0	Cell redox homeostasis	AgriGO-BP, Uniprot GO
***Glyma.13g361900***	19	3	6.667	37.14	Abscisic acid transport; response to ethylene; ATP catabolic process	Arabidopsis GO, Uniprot GO
*Glyma.14g127800*	19	3	8.822	18.72	ATP catabolic process	Uniprot GO
***Glyma.15g011900***	19	3	6.771	33.59	Abscisic acid transport; response to ethylene; ATP catabolic process	Arabidopsis GO, Uniprot GO
***Glyma.15g012000***	19	3	8.106	33.24	Abscisic acid transport; response to ethylene; ATP catabolic process	Arabidopsis GO, Uniprot GO
*Glyma.13g279900*	18	3	1.663	0	Jasmonic acid mediated signaling pathway	Arabidopsis GO
*Glyma.03g148300*	17	3	2.556	0	Response to gibberellin	Uniprot GO, Arabidopsis GO
*Glyma.03g112400*	16	2	0.611	NA		
*Glyma.02g005600*	16	3	2.498	6.31		
*Glyma.04g044900*	16	3	2.290	36.94	Photosynthesis; response to oxidative stress	Arabidopsis GO
*Glyma.06g045400*	16	3	2.250	4.37	Photosynthesis; regulation of root development; negative regulation of transcription, DNA-templated	Arabidopsis GO, Uniprot GO
*Glyma.10g180800*	16	3	2.372	20.14		
*Glyma.17g236200*	16	3	1.335	20.55	Negative regulation of transcription, DNA-templated; regulation of root development; response to abscisic acid	Arabidopsis GO, Uniprot GO
*Glyma.09g153900*	14	3	4.318	30.18	Glycolysis	AgriGO-BP, Uniprot GO
*Glyma.16g204600*	14	3	4.389	32.62	Glycolysis	AgriGO-BP, Uniprot GO
*Glyma.02g054200*	13	1	2.652	0	Methylation	Uniprot GO
*Glyma.19g013700*	13	2	2.764	32.28	Response to abscisic acid	Uniprot GO
*Glyma.07g153800*	13	3	4.777	0		
***Glyma.18g009700***	12	2	4.273	26.73	Gluconeogenesis; oxidation–reduction process; glucose metabolic process	Arabidopsis GO, Uniprot GO
*Glyma.02g268200*	12	3	1.844	1.84		
*Glyma.05g123900*	12	3	1.228	0		
*Glyma.05g124000*	12	3	1.304	0		
*Glyma.08g050400*	12	3	1.838	NA	Ethylene biosynthetic process	Arabidopsis GO
*Glyma.14g049000*	12	3	8.736	NA		
*Glyma.14g049200*	12	3	1.773	0		
*Glyma.02g009800*	11	3	1.645	0		
*Glyma.11g181200*	10	2	2.255	5.85		
***Glyma.07g153100***	10	3	3.256	20.67	Response to anoxia	Arabidopsis GO
*Glyma.12g149100*	10	3	2.385	0	Jasmonic acid mediated signaling pathway	Arabidopsis GO
*Glyma.01g206600*	9	2	1.517	5.91	Positive regulation of transcription, DNA-templated; regulation of transcription, DNA-templated	Arabidopsis GO
*Glyma.11g036500*	9	2	1.653	6.05	Positive regulation of transcription, DNA-templated; regulation of transcription, DNA-templated	Arabidopsis GO
*Glyma.17g145400*	9	2	1.383	2.43	Positive regulation of transcription, DNA-templated	Arabidopsis GO
*Glyma.09g276600*	8	1	1.786	3.30	Signal transduction	AgriGO-BP, Uniprot GO
*Glyma.14g203000*	8	1	3.251	0	Protein phosphorylation; response to pH	Arabidopsis GO
*Glyma.18g212700*	8	1	2.046	3.30	Signal transduction	AgriGO-BP
***Glyma.13g231700***	8	3	4.293	24.27		
*Glyma.05g150100*	7	1	0.532	0		
*Glyma.08g106900*	7	1	0.539	1.85		
*Glyma.11g180500*	7	1	2.800	33.04	Nuclear-transcribed mRNA poly(A) tail shortening	Arabidopsis GO
*Glyma.12g187400*	7	1	1.473	18.24	Defense response to bacterium; response to wounding	Uniprot GO
*Glyma.13g314100*	7	1	1.223	10.21	Nuclear-transcribed mRNA poly(A) tail shortening	Arabidopsis GO
*Glyma.13g005100*	7	2	1.603	16.83		
*Glyma.20g064500*	7	2	0.701	0		
*Glyma.04g240800*	7	3	2.998	0	Response to osmotic stress; cellular respiration	Arabidopsis GO
*Glyma.14g121200*	7	3	3.604	4.05	Response to osmotic stress; response to hypoxia	Arabidopsis GO, Uniprot GO
*Glyma.11g121900*	6	1	1.129	0		
*Glyma.13g239000*	6	1	5.001	0	Fatty acid biosynthetic process	Uniprot GO
*Glyma.04g190900*	6	3	2.889	3.01	Abscisic acid-activated signaling pathway	Arabidopsis GO
*Glyma.02g211600*	5	1	4.720	17.25		
*Glyma.10g195700*	5	1	3.781	0	Response to jasmonic acid	Uniprot GO
*Glyma.04g231400*	5	3	1.856	0		
*Glyma.06g133800*	5	3	1.767	0		
*Glyma.08g176300*	5	3	1.748	0	Response to osmotic stress	Uniprot GO
*Glyma.11g222600*	5	3	2.090	3.99	Response to abscisic acid	Arabidopsis GO
*Glyma.03g173300*	4	1	0.887	42.51	Regulation of root development; regulation of transcription, DNA-templated	Arabidopsis GO
*Glyma.07g105700*	4	1	1.375	8.28	MAPK cascade; ethylene biosynthetic process	Arabidopsis GO
*Glyma.09g172500*	4	1	1.443	3.85	Ethylene biosynthetic process; systemic acquired resistance, salicylic acid mediated signaling pathway	Arabidopsis GO
*Glyma.09g250700*	4	1	2.983	0	Dephosphorylation	Uniprot GO
*Glyma.12g093100*	4	1	1.787	30.92	Nuclear-transcribed mRNA poly(A) tail shortening	Arabidopsis GO
*Glyma.13g032100*	4	1	2.468	0		
*Glyma.15g045600*	4	1	2.655	0		
*Glyma.18g042100*	4	1	1.831	15.71	Protein ubiquitination	AgriGO-BP, Uniprot GO
*Glyma.19g174200*	4	1	0.805	25.18	Regulation of root development; regulation of transcription, DNA-templated	Arabidopsis GO
***Glyma.01g118000***	4	2	3.537	18.13	Response to anoxia	Arabidopsis GO
***Glyma.02g222400***	4	2	3.121	11.54	Glycolysis; response to cadmium ion	
*Glyma.13g270100*	4	2	2.011	5.43		
*Glyma.08g133600*	4	3	3.2		Response to osmotic stress	Arabidopsis GO
*Glyma.11g149900*	4	3	2.784	2.38		
*Glyma.11g255000*	4	3	2.822	0	Response to osmotic stress	Arabidopsis GO
*Glyma.04g092100*	3	1	1.16	11.90		
*Glyma.08g128500*	3	1	6.27	0	Cellular metabolic process	AgriGO-BP, Uniprot GO
*Glyma.17g164100*	3	2	3.264	0	Negative regulation of catalytic activity	AgriGO-BP
*Glyma.08g128100*	3	3	1.163	0		
*Glyma.12g150500*	3	3	2.312	1.79		
*Glyma.12g222400*	3	3	2.147	2.38		
*Glyma.01g037200*	2	1	1.785	0		
*Glyma.02g148200*	2	1	1.675	3.56		
*Glyma.05g108900*	2	1	2.098	3.44	Ethylene biosynthetic process	Arabidopsis GO
*Glyma.13g208000*	2	1	1.889	1.87		
*Glyma.17g158100*	2	1	2.109	3.44	Ethylene biosynthetic process; cell division	Arabidopsis GO
*Glyma.11g055700*	2	3	8.44	0	Abscisic acid biosynthetic process	Arabidopsis GO, Uniprot GO
*Glyma.17g174500*	2	3	12.853	0	Abscisic acid biosynthetic process	Arabidopsis GO, Uniprot GO
*Glyma.02g134500*	1	1	1.662	0		
*Glyma.03g015800*	1	1	0.831	6.58	Negative regulation of defense response	Arabidopsis GO
*Glyma.04g136600*	1	1	0.864	3.70		
*Glyma.06g100900*	1	1	1.206	4.36		
*Glyma.10g073600*	1	1	1.716	4.86	Anaerobic respiration	Arabidopsis GO
*Glyma.11g028200*	1	1	2.816	0	Cellular amino acid metabolic process	Uniprot GO
*Glyma.18g206000*	1	1	4.469	3.99	Defense response; abscisic acid-activated signaling pathway	AgriGO-BP, Arabidopsis GO
*Glyma.18g238100*	1	1	3.714	NA		
*Glyma.19g069200*	1	1	3.582	0	Protein dephosphorylation	Uniprot GO
*Glyma.19g213300*	1	1	4.844	0	Negative regulation of ethylene-activated signaling pathway	Arabidopsis GO
*Glyma.07g031400*	1	2	5.646	0	ATP catabolic process	Uniprot GO
*Glyma.08g199800*	1	2	5.023	0	Glycolysis	AgriGO-BP, Uniprot GO
*Glyma.09g149200*	1	2	2.164	0	Gibberellin biosynthetic process	Arabidopsis GO
*Glyma.13g250400*	1	2	4.118	0		
*Glyma.07g049900*	1	3	4.782	3.37	Glucan biosynthetic process	Uniprot GO
*Glyma.16g018500*	1	3	4.614	3.37	Glucan biosynthetic process	Uniprot GO
*Glyma.20g218100*	1	3	4.614	0	Glucan biosynthetic process	Uniprot GO

*N*^*pw*^ number of enriched pathways, *N*^*method*^ number of pathway analytic approaches, *NA* not available, *GO* gene ontology.

^a^Z-score value was calculated using a binomial proportions test.

Significant values are in bold.

**Figure 7:**
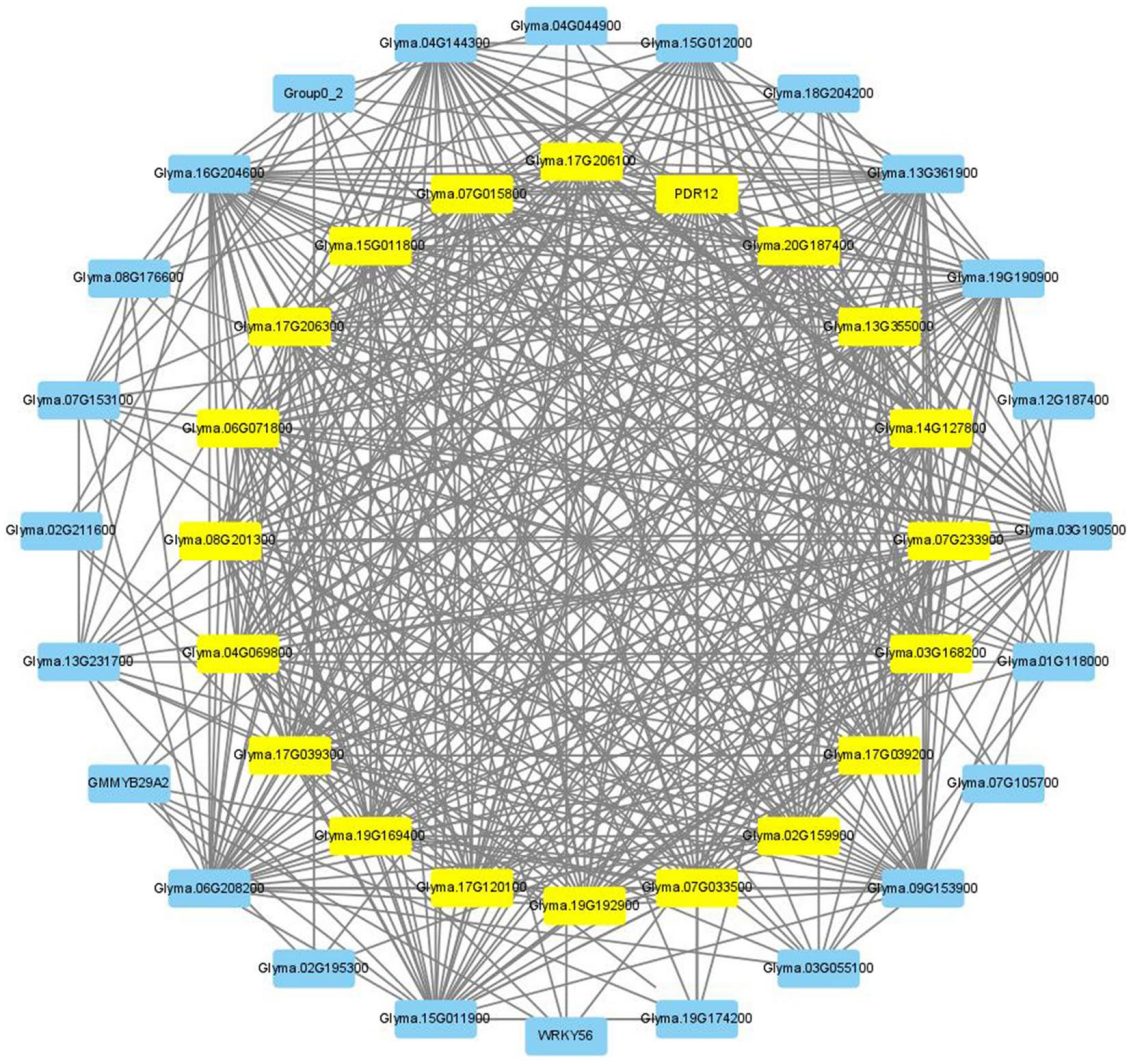
The gene network analysis of FTgenes associated with enriched pathways in the hypergeometric test. A total of 77 FTgenes were selected from 24 significantly enriched pathways in hypergeometric tests in four time-point databases to construct the gene network. Software Cytoscape v3.9.0 (https://cytoscape.org/) was used to create image.

We selected 77 FTgenes from 24 significantly enriched pathways reported in the hypergeometric test to compute node edges in SoyNet and construct a gene network in Cytoscape. As a result, 103 genes (74 FTgenes and 29 intermediate genes) were retained, with degree values ranging from 0 to 35 (the average degree was 9.68). For simplicity, we further grouped 60 genes having a row degree of centrality into a node (named as Group0_2), and included the node with the remaining 43 genes (15 FTgenes and 28 intermediate genes) to form a gene network (Fig. 7; Sheet 2 in Supplementary Table 1). Of those, 20 genes (highlighted in yellow) having higher degree values between 20 and 30, with an average degree of 28.2, demonstrated to interact with each other more closely in the gene network. Among them, one Ftgenes (*Glyma.14g127800*) is mainly related to plant hormone transport that contributes to flooding-tolerant responses.

**Figure 8:**
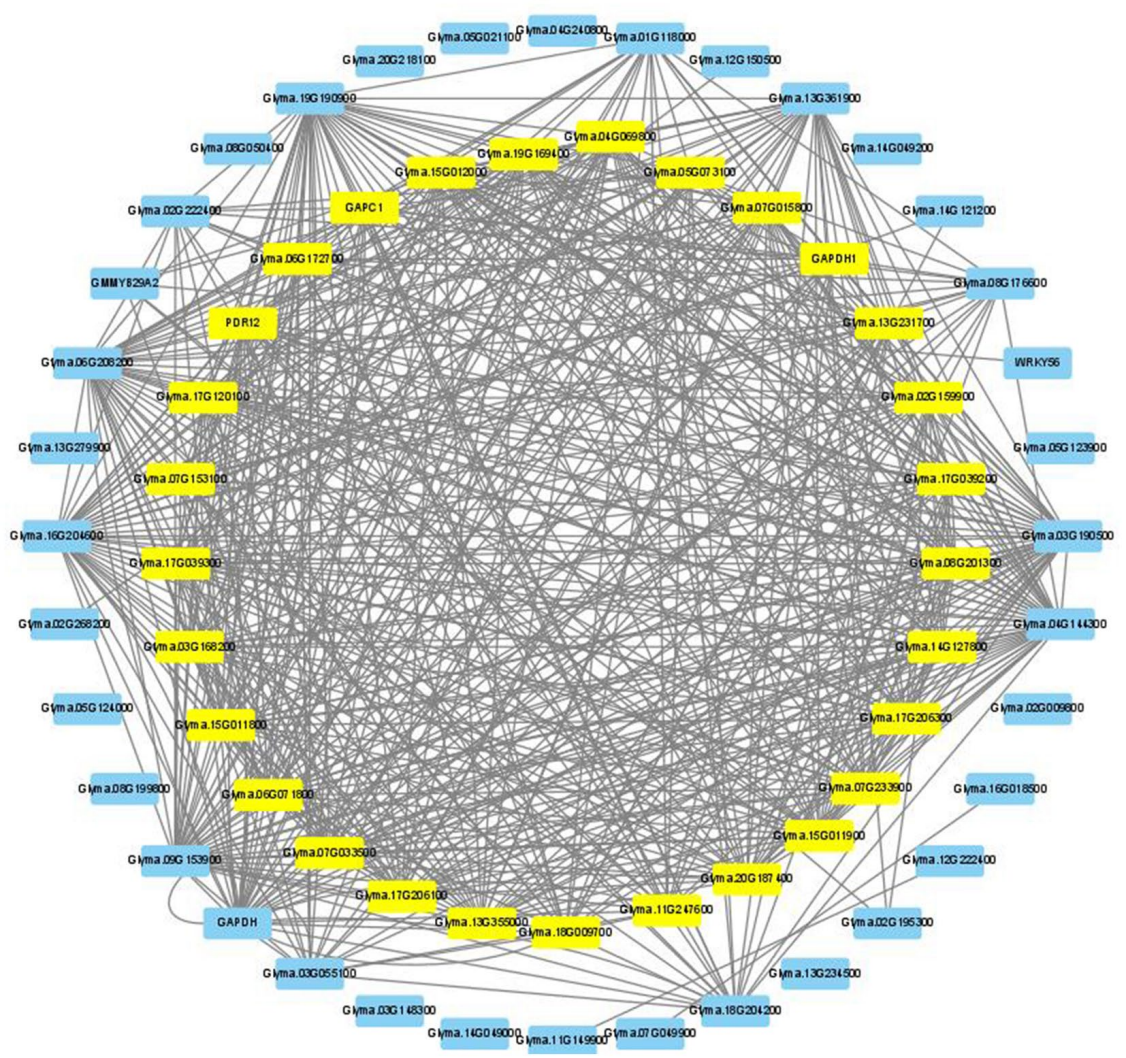
The gene network analysis of FTgenes associated with enriched pathways in SUMSTAT test. A total of 34 FTgenes were selected from 5 significantly enriched pathways in SUMSTAT in four time-point databases to construct the gene network. Software Cytoscape v3.9.0 (https://cytoscape.org/) was used to create image.

Another 34 Ftgenes from 5 significantly enriched pathways reported in SUMSTAT were being computed edges in SoyNet, resulting in 65 genes (32 Ftgenes and 33 intermediate genes), with degree values ranging from 0 to 71 (the average degree was 17.94). We further constructed a gene network in Cytoscape (Fig. 8; Sheet 3 in Supplementary Table 1), and observed that 29 genes (highlighted in yellow) were highly connected to form a dense module, with higher degree values between 20 and 29 (the average degree was 25.5). Among them, 6 Ftgenes are mainly related to signal transduction (*Glyma.15g011900, Glyma.15g012000*), plant hormone transport (*Glyma.14G127800, Glyma.18G009700*), and enzyme catalytic activity (*Glyma.13G231700, Glyma.07G153100*).

The above results show closely connected PPIs in the soybean interactome by entering the 144 FTgenes, 24 enriched pathways at all four-time points (3, 6, 12, and 24 h) in the hypergeometric test, and 5 enriched pathways at all four-time points in the SUMSTAT method, respectively. These genes were first compared to 23 (Fig. 6), 20 (Fig. 7), and 29 (Fig. 8) selected important genes (including the FTgenes and the intermediate genes) to examine their topological characteristics. Our results showed that these important genes had higher degree values in all comparisons, suggesting high degree of centrality. These FTgenes (103, 77, and 34 FTgenes) were further compared to the intermediate genes (70, 29, and 33 genes) and the remaining genes (14,599, 16,911, and 18,993 genes), respectively. We found that the FTgenes and the intermediate genes in the corresponding gene network more frequently received small p-values at all four-time points in gene expression datasets.

**Figure 9:**
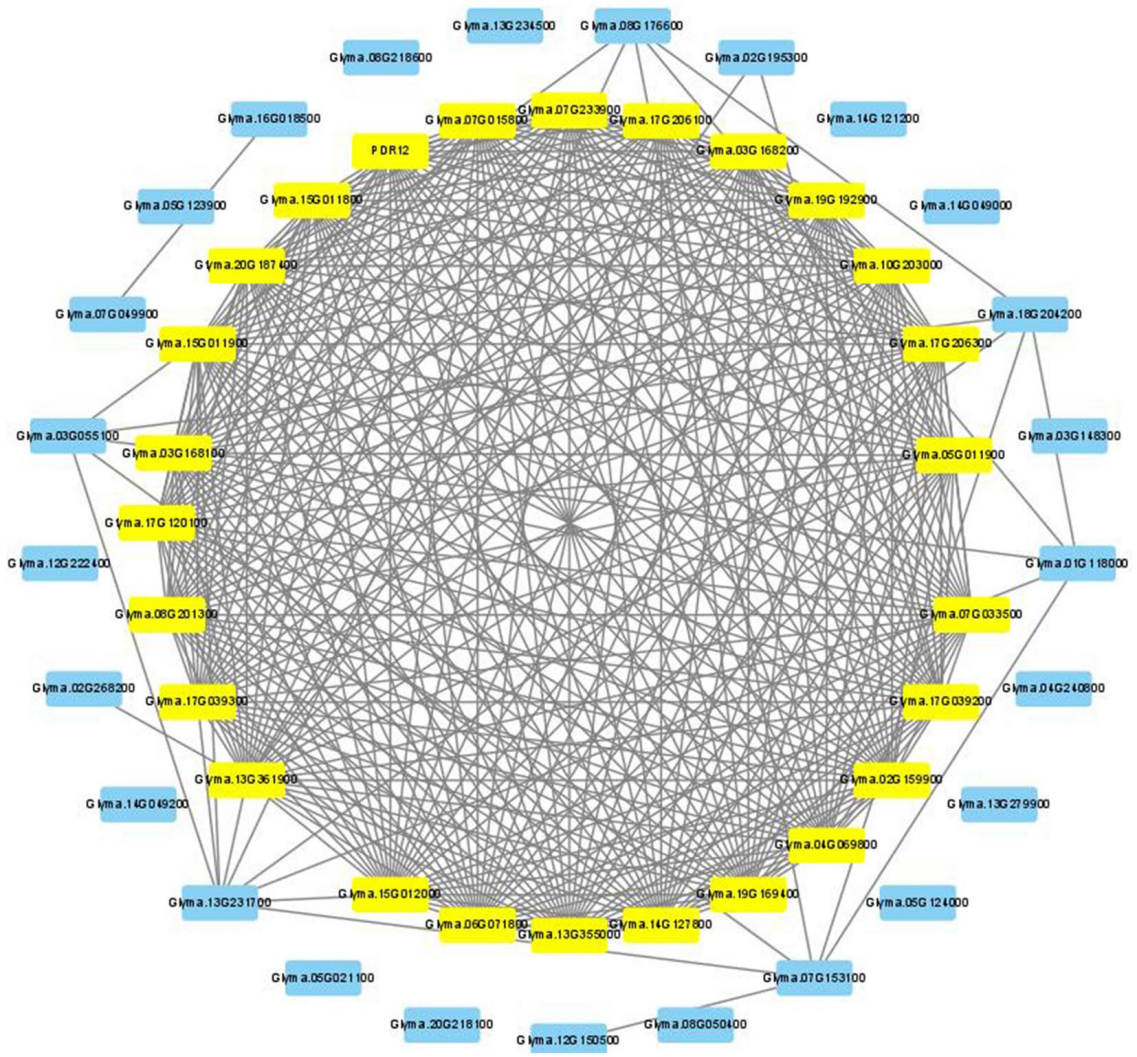
The gene network analysis of FTgenes associated with enriched pathways in hypergeometric test and SUMSTAT. A total of 25 FTgenes were selected from 5 enriched pathways overlapped in both hypergeometric test and SUMSTAT to construct the gene network. Software Cytoscape v3.9.0 (https://cytoscape.org/) was used to create image.

To further explore the key FTgenes, we selected 25 FTgenes from 5 significantly enriched pathways that overlapped in both the hypergeometric test and the SUMSTAT method to compute node edges in SoyNet and construct a gene network in Cytoscape. As a result, a gene network containing 25 FTgenes and 26 intermediate genes was obtained (Fig. 9; Sheet 4 in Supplementary Table 1), with degree values ranged from 0 to 28 (the average degree was 13.68). Of them, 26 genes (highlighted in yellow) were closely linked, having higher degree values between 20 and 30, with an average degree of 25.3. Among them, four FTgenes (*Glyma.13g361900*, *Glyma.15g012000*, *Glyma.15g011900*, and *Glyma.14g127800*) exhibited higher degree values ranged between 26 and 28, with an average degree value of 26.3. The four FTgenes are mainly related to signal transduction (*Glyma.13g361900, Glyma.15g011900, Glyma.15g012000*) and plant hormone transport (*Glyma.14g127800*) to play key roles in flooding-tolerant responses in soybean.

**Figure 10:**
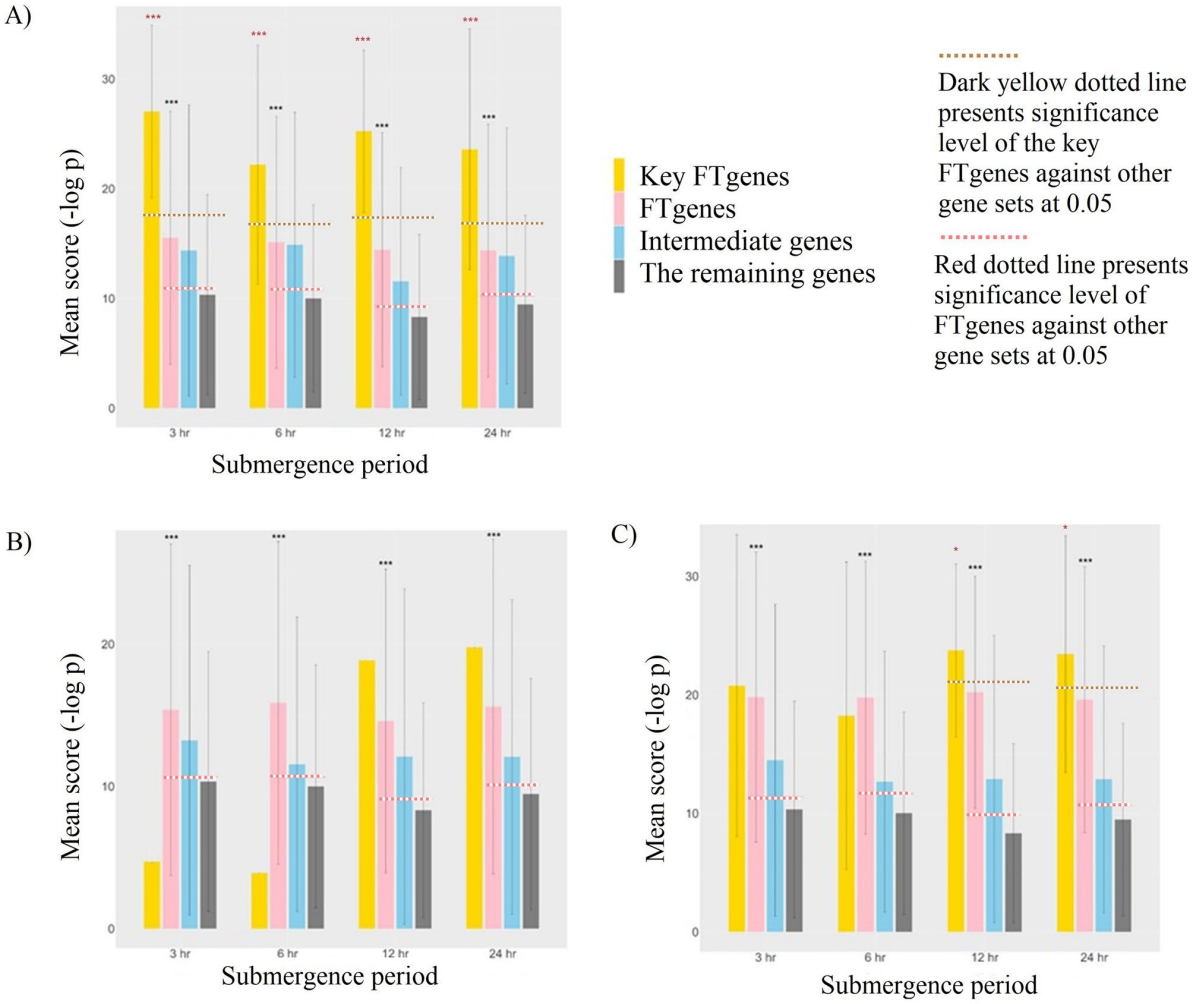
Validation study of the FTgenes (and the key FTgenes) compared to the intermediate genes and the remaining genes using an independent RNA-seq data. (**A**) 103 FTgenes were selected from enriched pathways. (**B**) 77 FTgenes were selected from 24 significantly enriched pathways in the hypergeometric test. (**C**) 34 FTgenes were selected from 5 significantly enriched pathways in SUMSTAT. The dark yellow and red dotted line presents the significance level of the key FTgenes and the FTgenes against other gene sets at 0.05, respectively. The red star represents that the key FTgenes have a significantly higher mean score than all other gene sets. The dark grey star represents that the FTgenes have a significantly higher mean score than the remaining genes. The threshold of statistical significance level: *p-value < 0.05, **p-value < 0.01, ***p-value < 0.001.

**Figure 11:**
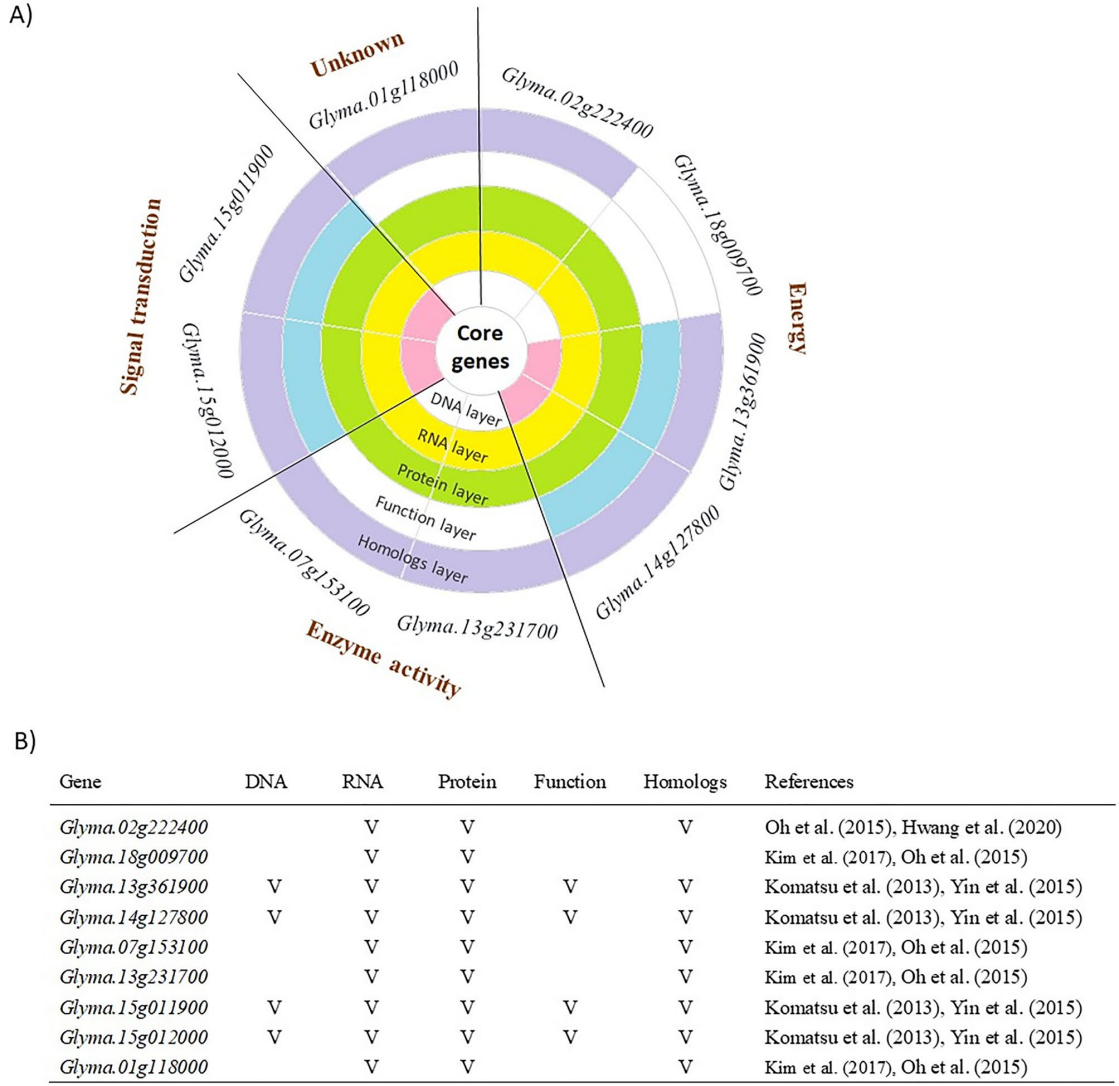
Summary information of nine key FTgenes. (**A**) The sunburst chart of the nine key FTgenes and their involved gene functions. (**B**) The evidence was obtained from different layers for the nine key FTgenes.

More importantly, all these FTgenes in the corresponding gene networks had significantly larger mean scores (or smaller p-values) in the corresponding gene expression datasets (p-values < 0.001) compared to the remaining genes (Fig. 10). Similar scenarios were also observed in the intermediate genes, although they did not reach significance level at 0.05. In particular, we further selected 8, 1, and 6 key FTgenes from the corresponding gene network, respectively, and found that these key FTgenes significantly outperformed all gene groups (Step 5 in Figs. 1, 10). The nine key FTgenes (Fig. 11A) were involving with signal transduction (*Glyma.15g012000* and *Glyma.15g011900*), energy (*Glyma.02g222400*, *Glyma.18g009700*, *Glyma.13g361900*, and *Glyma.14g127800*), enzyme activity (*Glyma.07g153100* and *Glyma.13g231700*), and unknown function (*Glyma.01g118000*), which were significantly related to abscisic acid transport and terpenoid transport.

**Figure 12:**
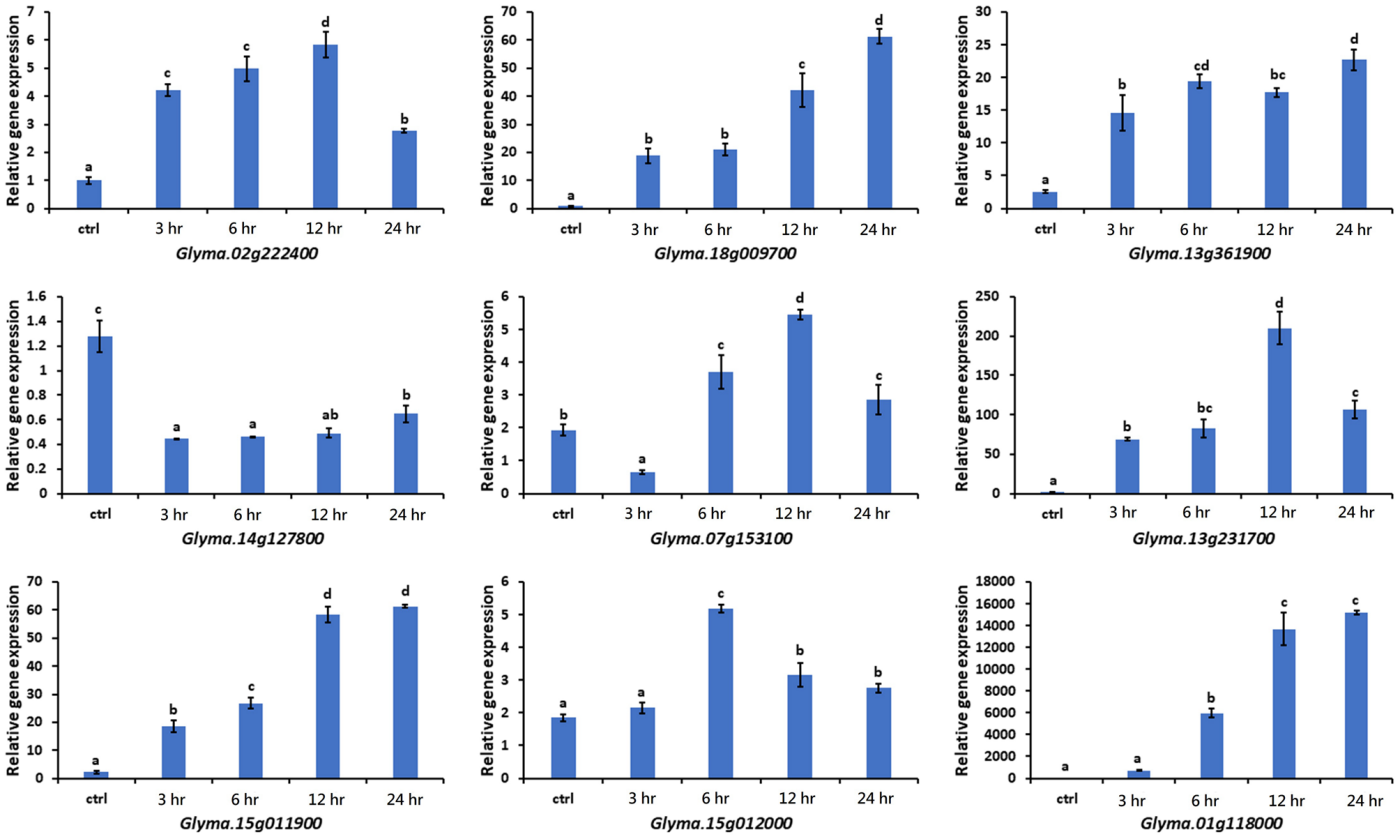
Relative gene expression of 9 key FTgenes in soybean root under flooding stress. Actin was used as a reference gene, and untreated was used as the reference condition (ctrl). Error bars indicate ± standard error. Different lowercase letters above columns indicate significant differences at p < 0.05.

To validate the flooding stress responses in the plant cell, a real-time quantitative reverse transcription polymerase chain reaction (qRT-PCR) was used to measure the level of the nine key FTgenes expressions in soybean root under flooding stress (Step 6 in Figs. 1, 12). Our results revealed that four energy involved genes (*Glyma.02g222400*, *Glyma.18g009700*, *Glyma.13g361900*, and *Glyma.14g127800*) were significantly upregulated from 3 to 24 h except for *Glyma.14g127800* which showed downregulation in all conditions compared with the control (i.e. untreated condition). In the enzyme activity involved genes (*Glyma.07g153100* and *Glyma.13g231700*), the highest expression was found at 12 h after treatment. For those of signal transduction involved genes, the transcript level of *Glyma.15g011900* and *Glyma.15g012000* was significantly higher than the control from 3–24 h to 6–24 h, respectively. Interestingly, the *Glyma.01g118000* which is an unknown function gene exhibited around 330–7000 times higher expression level than the control, and when compared with the other, this gene also has the highest relative gene expression.

## Discussion

Understanding genetic backgrounds and molecular mechanisms underlying flooding-tolerant responses is imperative for soybean breeding. However, the success in identifying candidate genes for flooding-tolerant responses in soybean has been limited because of the complex nature of abiotic stresses. The present study introduced systems biology methods using pathway enrichment analysis (both competitive and self-contained approaches were considered) and gene network analysis to evaluate the joint effects of multi-genes (in this context, FTgenes) within annotated GO pathways. Most importantly, the FTgenes^36^ (Fig. 2) used in this study were prioritized from multiple OnO databases integrated from experimental and computational studies that have been made available in the last decades. In particular, several data-ensemble approaches were performed, including data cleaning, data harmonization, data heterogeneity, and data mapping, to remove unwanted data and inaccurate data. Through the process of gene prioritization, the uncertainties, noise, biases, and false positives raised from the data itself and statistical approaches could be reduced effectively.

Systems biology often requires sophisticated computational models and simulations to understand the larger picture of the biological systems by studying interactions among a set of candidate genes^59^. Integrative pathway and network analysis marry the idea of mathematical graph theory and data-driven approach (e.g. multiple omics data, OnO data integration) to efficiently uncover the genotype–phenotype relationship at the systems level by integrating knowledge of gene regulation and function. In an attempt to integrate OnO data with mathematical graph theory, we introduced an integrative pathway and network approach to construct a comprehensive view of the biological mechanisms for flooding-tolerant responses in soybean. To the best of our knowledge, this is the first work on the pathway and network analyses using candidate genes prioritized from multiple OnO data integration algorithms. Our results reveals novel molecular pathways and functional relationships of the FTgenes to better understand their biological implications in the regulatory system for further validation.

Skewness is widely used to measure the degree of asymmetry in expression data. Expression skewness can identify novel molecular pathways and key genes via the systems biology approaches (e.g. enrichment pathway analysis and functional network analysis), which is a valuable way to capture meaningful outliers (i.e. the greatest variation between samples with and without submergence) and asymmetrical behavior in the whole genome expression dataset^60^. Our results demonstrated a high degree of skewness (Fig. 3) that was appropriate for pathway and network analyses. In addition, our results may provide valuable insights into exploring mechanisms underlying flooding-tolerant responses in soybean.

In the studies of crops, pathway analysis is merely used for the exploration of candidate genes focusing on specific traits^58,61^. In general, pathway analysis can be distinguished into two different approaches, the competitive and the self-contained, according to their null hypothesis^48^. In practical applications, however, two different approaches often generated inconsistent results^62^ due to distinct null hypotheses. The competitive methods can potentially exclude confounding effects and provide biological relevance to the analysis^63^. The self-contained methods have the greater power to identify feature-set (i.e. GO pathways), and the outcomes are highly reproducible^64^. Both approaches have their strengths and limitations. Therefore, a suitable way to gain better insights into the data is to perform the competitive and the self-contained approaches simultaneously for feature-set (i.e. GO pathways) testing. This could reduce the likelihood of false-positive results and gain biological relevance to the analysis.

In this study, we identified 36 overrepresented GO pathways (Tables 1, 2, 3) in the independent RNA-seq databases of submergence treatments in soybean. The most frequently shared FTgenes among enriched pathways were *Glyma.02g195300* (functioning in 23 pathways), *Glyma.05g021100* (functioning in 20 pathways), *Glyma.08g218600* (functioning in 20 pathways), and *Glyma.14g102900* (functioning in 20 pathways), which were found in two or more pathway-based methods (Table 4). Many of these FTgenes (6, 18, 10, and 12 FTgenes in 3, 6, 12, and 24 h, respectively) were not significantly overrepresented at the single gene-level in the RNA-seq databases (Fig. 3); however, they were enriched (p-values < 0.001) with flood-tolerant responses at the systems level using pathway-based analytic approaches. For instance, *Glyma.17g236200*, *Glyma.19g013700*, and *Glyma.11g180500* gene did not reach genome-wide significant association, but were found at the systems level in our approaches. In particular, Arabidopsis GO and Uniprot GO databases provide opportunities to access a better understanding of how these FTgenes participate in flooding activities. Under flooding conditions, the *Glyma.17g236200* gene regulates root development to prevent from wounding, and the *Glyma.19g013700* gene mediates the transpiration efficiency by regulating ABA to control stomata closure. In further, the *Glyma.11g180500* gene participates in RNA regulation, producing factors to control where plant hormones should work. Evidence from previous studies confirmed the roles of these important FTgenes and pathways identified in this study for the complex mechanisms of flooding-tolerant responses in soybean. These findings indicate that systems biology methods can boost the power to reveal the potential roles of FTgenes in uncovering the molecular mechanisms and biological novelties for studying flooding-tolerant responses in soybean.

The hypothesis and the model of different categories of pathway analysis are distinct; hence, the results are also different. In this study, we compared the results across the hypergeometric test, the SUMSTAT method, and the SUMSQ method. In total, 27, 14, and 1 enriched pathway(s) were identified in the hypergeometric test (Table 1, Fig. 4), the SUMSTAT method (Table 2, Fig. 5A), and the SUMSQ method (Table 3, Fig. 5B), respectively. The three methods found only one pathway, ‘response to hypoxia’ in all four-time points (3, 6, 12, and 24 h) of gene expression data. Under flooding conditions, the response to hypoxia begins with low-oxygen stimulation, followed by activates the transcription of plant hormone genes. Plant hormones, such as ABA, ethylene, and salicylic acid, are involved in participating in roots recovery^65^. Of which five pathways were consistently reported by both the competitive and the self-contained approaches, even a more stringent threshold was applied to correct for multiple testing. The ‘systemic acquired resistance, salicylic acid mediated signaling pathway’ is responsible for regulating the biosynthesis, the perception, and the signal mediating^66^. When a plant suffers from flooding, hydrogen peroxide begins to express in roots to remove some harmful chemicals from flooding stress, and salicylic acid mediates a series of signals in producing hydrogen peroxide^66,67^. Evidence shows the important fact that the regulation of hydrogen peroxide interacts with salicylic acid by signaling series forms to eliminate fatal chemicals in roots cell under flooding stress^66–68^. Thus, ‘regulation of hydrogen peroxide metabolic process’ and ‘systemic acquired resistance, salicylic acid mediated signaling pathway’ are evidenced to be linked to flooding-tolerant responses in soybean. Besides, rice was also evidenced to be involved with these two pathways under flooding stress^69,70^. In soybean roots, cell wall and aerenchyma will swell under flooding. The response to wounding in roots leads to many salicylic acid signals activating and interacting with other plant hormones in order to restore the wounds^71^. After wounding, the soybean’s adventitious roots will grow against hypoxia environments. The energy from glycolysis and pyruvate-phosphorylation is consumed when soybean grows adventitious. Evidence showed that ‘response to wounding’ and plant hormone-related pathways may play key roles in flooding-tolerant responses in soybean. In addition, the gluconeogenesis and glycolysis, which can synthesize or degrade carbohydrates, make crops gain and store adequate ATPs in order to get more energy^20–22,33^. All the evidence suggested that ‘glycolysis’, ‘gluconeogenesis’, ‘pyruvate decarboxylase activity’, ‘abscisic acid mediated signaling pathway’, and ‘regulation of hydrogen peroxide metabolic process’ were found to be linked to flooding-tolerant responses in soybean, which were in line with the previous studies^20–22,33^. All these pathways mentioned above play key roles in the physiological mechanisms underlying flooding-tolerant responses. Our results demonstrated that the pathways we found differ considerably between distinct types of pathway analyses. Hence, combining distinct pathway-based analyses with considering different hypotheses and models can provide comprehensive, precise, and reliable results.

Ethylene is important to protein phosphorylation in the mechanisms of the initial stage of flooding stress, especially in root tips. Evidence shows that roots recovery needs more ATP to provide energy and protein phosphorylation to develop the cell tissue^25,28,29,37^. At the initial stage of flooding, root cells are stimulated by ethylene, and a series of mediated signaling produce more ethylene. The evidence proves that ‘response to ethylene stimulus’, ‘ethylene biosynthetic process’, and ‘ethylene mediated signaling pathway’ are important to flooding stress.

Our results also showed 22 (Table 1, Fig. 4) and 9 (Table 2, Fig. 5A) enriched pathways specific to the hypergeometric test and the SUMSTAT method, respectively. Without comparing the results of two different approaches, we might obtain false-positive and false-negative results. For instance, two pathways, ‘glycolysis’ and ‘gluconeogenesis’, were evidenced^19–21,33^ and found in the SUMSTAT approach but not in the hypergeometric test, and hence they are false-negative results; the ‘abscisic acid mediated signaling pathway’ pathway was evidenced^22,25,28,33^ and reported in the hypergeometric test but not in the SUMSTAT, and hence it is a false-negative result. Two pathways, ’response to chitin’ and ‘response to fungus’, were significantly enriched in the hypergeometric test, but did not reach the significance in the SUMSTAT approach. Besides, the two pathways were evidenced to be related to biotic stress^72^. Hence, the two pathways might be false-positive results or novel findings that need further validation. Again, combining the competitive and the self-contained methods is a promising approach to better understanding a given candidate genes for a trait of interest.

Benefits from combining advantages of pathway enrichment analysis and network analysis, our study not only discovers new novelty about the flooding mechanisms in soybean but also captures more information of biological systems. For instance, We finally selected nine key FTgenes, four of these genes (*Glyma.13g361900, Glyma.14g127800, Glyma.15g012000*, and *Glyma.15g011900*) were recorded in DNA, RNA, protein, function, and homologs layer; one gene (*Glyma.18g009700*) was recorded in RNA and protein layer; and four genes (*Glyma.02g222400*, *Glyma.07g153100*, *Glyma.13g231700*, and *Glyma.01g118000*) were recorded in RNA, protein, and homolog layer^36^ (Fig. 11B). These key genes may play important roles in coordinating physiological mechanisms under flooding-tolerant responses in soybean.

The systems biology framework proposed in this study demonstrated the power in identifying the nine key FTgenes in a rigorous and efficient manner. To validate the 9 key FTgenes, in planta FTgenes expression analysis was performed. Our qRT-PCR results (Fig. 12) revealed that eight key FTgenes were upregulated and one FTgene was downregulated after exposed to flooding stress. The results demonstrated the unique and differential response of soybean leaf tissue under flooding, offering the evidence of the real response to flooding in genetics and molecular biology. Our results can be supplied as a good foundation for the gene function analysis underlying flooding-tolerant responses in further work.

Although systems biology takes advantage of a comprehensive and systematic understanding of the FTgenes in flooding-tolerant responses in soybean, there still are some limitations and considerations in this study. First, pathway and network analyses were built on the basis of gene and pathway annotation completeness. In the application of scientific research, the research team of GO and PlantRegMap updates the databases and maintains the website annually. It ensures the databases are complete, accurate, and persuasive. Second, the accuracy of pathway and network analyses relied on the accuracy and the completeness of the FTgenes. Fortunately, our FTgenes were selected from a comprehensive framework consisting of omics and non-omics data integration and gene prioritization algorithm. Several data quality control processes were done during the data-ensemble step to effectively reduce potential uncertainties, noise, and false positive results. Although our FTgenes are informative, more validation experiments are required.

Flooding-tolerant responses are a quantitative trait regulated by polygenes; thus, many traditional single-marker methods, such as association mapping, linkage mapping, and genome-wide association study, have no power to uncover the whole picture of how these genes interact with each other to regulate traits. Our proposed systems biology framework can efficiently integrate gene information with annotated GO database biologically to boost the power of identifying key FTgenes and their underlying molecular pathways or mechanisms. This provides an opportunity to better understanding complex flooding-tolerant responses that should be noted. These findings present a wealth of information for future validation.

## Methods

We developed an integrative systems biology framework to explore insights into the FTgenes underlying flooding-tolerant responses in soybean. Six-step pipelines (Fig. 1) were proposed to select the key FTgenes, including the GO annotations filtering, gene-wise statistic scores calculation, pathway enrichment analysis, functional network analysis, validation study, and the key gene selection. Detailed methods and materials used in this study are described below.

### Candidate genes for flooding-tolerance (FTgenes)

We previously proposed a comprehensive multiple OnO data mining, integration, and prioritization framework^36^. All genetic data (SNPs, genes, SSRs, QTLs) and bioinformatics information (trait index, variety, biochemical, statistical values) that were relevant to flooding-tolerant responses in soybean were collected and defined as a flooding-tolerance gene pool (containing 36,705 genes). These OnO data were integrated from multidimensional data platforms, including association mapping and GWAS, linkage mapping, gene expression, pathway regulatory, network analysis, protein–protein interaction, proteomic analysis, and model plants. Through the systems biology framework, a total of 144 prioritized FTgenes (Fig. 2), based on the cut-off score of 42, were selected from the gene pool^36^. The FTgenes were defined to be significantly associated or enriched with flood-tolerance or flood-response after flooding treatment (i.e., submergence) was conducted during the germination and vegetative growth stages of soybean. The study framework and the prioritized results, the data of which are used here, are provided elsewhere^36^.

### Gene expression dataset and gene-wise statistic values

The gene expression dataset (whole genome expression database) of soybean seedling submergence was accessed through the database of Genotypes and Phenotypes (dbGaP, https://www.ncbi.nlm.nih.gov/gap/) that was published by Lin et al.^58^. They used cultivar Qihuang 34, a flooding-resistant variety, for submergence experiments, and recorded gene expression changes in roots after 3, 6, 12, and 24 h of submergence treatments. All four-time periods of RNA expression data were obtained from the dbGaP repository. We used p-values, of which genes under the null hypothesis of no differential gene expression, to present gene-level statistic values of flooding-tolerance in soybean. To obtain gene-level significance, we used 10-based logarithms to transform p-values into gene-wise statistic scores to capture information for gene expression changes in roots flooded after 3, 6, 12, and 24 h.

### Pathway annotations

To perform mapping for functional pathway analysis, we used GO^73,74^ (http://geneontology.org/) annotations. GO-based functional annotation in soybean contains 4896 terms covering 48,606 unique genes, mapped in the Williams 82 reference genome version 2 (*Glycine max* Wm82.a2.v1). These annotated GO gene sets (i.e. pathways) systematically provide a standard catalogue to classify functional genes into biological functions and molecular mechanisms. Pathways with overly limited information (< 6 genes) were removed, as well as substantially large (> 1500 genes) pathways. As a result, a total of 2926 pathways, which consist of 916 cellular components, 762 biological processes, and 1248 molecular functions, remained for pathway analysis. In pathway analysis, we used the negative logarithm of these 2926 pathways’ p-values as our statistic.

### Statistical methods for pathway enrichment analysis

We utilized two different strategies, the competitive method, and the self-contained method^47^, to test for significantly enriched pathways for the trait of flooding-tolerance in soybean. Three statistical methods, including the hypergeometric test (competitive method), SUMSTAT, and SUMSQ (self-contained methods), were used to discover the significance of enriched pathways. The former method compares two gene sets in terms of association with a phenotypic trait based on a statistical probability model, and the latter two methods only test the association between a phenotypic trait and genes in pathways.

The hypergeometric test, assuming an experimentally-derived gene list is randomly conditional on a fixed pathway, is a widely utilized competitive method for pathway analysis^75^. The null hypothesis of the test is that genes in a pathway are more strongly associated with the phenotypic trait than those outside the pathway. The main idea of the test is to sample randomly, without replacement, from a finite population, calculating the statistic of characteristic (here is flooding-tolerance) of interest. Hence, this method aims to test whether annotated pathways (i.e. biological functions or processes), which are functionally related, are enriched or over-represented in a list of important genes (i.e. FTgenes) with the trait of interest. The p-value can be computed by

$$\mathrm{p-value }= \sum_{x=g}^{S}\frac{\left(\begin{array}{c}S\\ x\end{array}\right)\left(\begin{array}{c}L-S\\ M-x\end{array}\right)}{\left(\begin{array}{c}L\\ M\end{array}\right)},$$

where *L* is the total number of genes in a finite population, *M* is the size of important genes, *S* is the number of genes in a specific pathway, *x* is the number of important genes in a specific pathway, and *g* is the number of genes in *M*.

The idea of the self-contained methods is to use permutations to generate a huge number of null distributions. We compared genes in a specific pathway with random sets sampled from the hull distributions and calculated an empirical p-value for pathway analysis. The tests ignored genes not in the pathways. The present study applied two self-contained methods, SUMSTAT and SUMSQ^76^. Under the null hypothesis (the pathway is unrelated to the trait), we tested whether the observed gene set (i.e. pathway) outperforms the random gene sets generated by permutations. The enrichment score (ES) calculation of SUMSTAT and SUMSQ can be expressed as

$$(\mathrm{SUMSTAT})\quad \mathrm{ ES_{SUMSTAT} }=\sum_{i=1}^{S}{t}_{i},$$

$$\left(\mathrm{SUMSQ}\right)\quad \mathrm{ ES_{SUMSQ }}=\sum_{i=1}^{S}{t}_{i}^{2},$$

where *t*_*i*_ is the *i*-th value of the statistic (in this context, expression metrics e.g. p-value, fold-change) of FTgenes, and *S* is the number of genes in a specific pathway.

The analysis pipelines of SUMSTAT and SUMSQ consist of calculating the statistics ES of observed gene sets of soybeans, random permutations of statistics calculated from gene expression data, calculating permuted ES and association p-value. The ES represents association signals for each of annotated pathways, and the calculation of ES_SUMSTAT_ and ES_SUMSQ_ is to sum over all statistics and all squared statistics of a gene set (i.e. GO pathway) containing *S* FTgenes, respectively. We randomly shuffled the statistics calculated from gene expression data for each pathway and followed the same receipt above to calculate a permuted ES. Then, we normalized the ES by subtracting the mean of permutated ESs, and divided it by the standard deviation of permuted ESs. Finally, we calculated empirical p-values by comparing the observed ES and the permuted ES in 10,000 permutations for all pathways.

### Functional gene network analysis

A graphical model of a network composes of nodes and edges. Nodes can be defined as genes, proteins, metabolites, and annotated pathways. Edges are typically presented by connections between nodes. In network analysis, the degree is the most widely used measure to describe the connections of the nodes in a network. In this study, we defined nodes as the FTgenes, and calculated edges using the sum of the log-likelihood score in SoyNet functional gene network tool (https://www.inetbio.org/soynet/Network_nfm_form_conv.php). For detailed steps of network links calculation, please refer to Berger et al.^77^ and Kim et al.^78^. We further used Cytoscape v3.9.0^79^ to integrate molecular interaction network data to visualize the graphical model of the network.

### Multiple testing correction

To account for multiple testing problems in pathway analysis, we applied both the Benjamini–Hochberg correction method^80^ and the Bonferroni correction method to balance false positive and false negative results. The procedure controls the false discovery rate at 0.05 level in the current study, assuming p-values are independently distributed under the null hypothesis. Only pathways reaching genome-wide significance threshold of p-value less than 1.00 × 10^–4^ were considered significantly enriched.

### Validation for the key FTgenes

Soybean seeds of Chiangmai 60 cultivar were obtained from Thanya Farm Co., Ltd., Nonthaburi, Thailand. The seeds were surface-sterilized in 1% sodium hypochlorite and rinsed with distilled water 3 times. The seeds culture and stress conditions were done following Lin et al.^58^ with some modification. Ten seeds were sown on the sandy soil in a plastic pot (240-mm length × 240-mm width × 190-mm depth). A total of eight pots were sowed. Five seedlings soybean with the same size were retained in the pot, when two true leaves were fully unfolded (~ 8 days), the seedlings pot was transferred into new plastic containers filled with water. The samples were collected at 3, 6, 12, and 24 h, respectively. The untreated plants were used as the control. The root was collected and immediately frozen in liquid nitrogen for RNA extraction.

Total RNA was isolated from root and shoot with TRIzol reagent according to the manufacturer’s protocol. Subsequently, 5 µg of the total RNA was mixed with 500 ng of oligo(dT)_18_ and 200 U Superscript™ III reverse transcriptase (Invitrogen), and the mixture was reverse transcribed at 50 °C for 60 min. The real-time PCR was done following the manufacturer’s protocol of the Luna^®^ Universal qPCR Master Mix (NEB) with the gene-specific primers listed in Supplementary Table 2. After the PCR had finished, the PCR specificity was examined using 2% agarose gel and the relative gene expression ratios were calculated using the 2^−ΔΔCT^ method with untreated plants cDNA as the reference sample and actin as the reference gene. All experiments were done in biological triplicates.

To validate the significant differences between the transcript quantities of FTgenes under flooding stress, statistical analysis was performed using one-way ANOVA followed by Tukey’s HSD test method facilitated by the IBM SPSS statistics software. p-values less than 0.05 were considered as statistically significant difference.

## Conclusions

This study shed new light on the effectiveness of the systems biology framework based on the FTgenes selected from the integrated OnO data and gene prioritization algorithm to uncover the mechanisms behind flooding-tolerant responses in soybean. We proposed a computational systems biology pipeline to discover enriched pathways and nine key genes that were real responses to flooding stress in our qRT-PCR experiments. This work suggests that the integrative pathway and network framework at systems biology level can be a good foundation for key genes discovery and gene function analysis for further work. In addition, this pipeline can minimize potential uncertainties and false positives and gain valuable insights into mechanisms underlying flooding-tolerant responses in soybean. The framework presented in this work can be applied to other complex traits in important crops.

## Supplementary Information

Supplementary Table 1.

Supplementary Table 2.

## Data Availability

The raw RNA-seq data of the whole genome gene expression dataset of soybean seedling submergence can be accessed in the NCBI Sequence Read Archive (SRA), and the accession number is SRP181976. The data of the flooding-tolerance genes (FTgenes) presented in the study are deposited in the DRYAD repository, accession number for a unique digital object identifier (DOI): 10.5061/dryad.dv41ns229. The dataset of the FTgenes is available at https://datadryad.org/stash/share/yfjZHzx6Oal5UyUr87EISoC6txczBChObdEOYAwSbTE. Soybean seeds were obtained from Thanya Farm Co., Ltd., Nonthaburi, Thailand.
