# Supplementary material for: Integrative pathway and network analysis provide insights on flooding-tolerance genes in soybean
Source: Sci Rep. 2023 Feb 3;13:1980. doi: 10.1038/s41598-023-28593-1 (PMC9898312; doi:10.1038/s41598-023-28593-1)
Supplement: Supplementary file 2 — Supplementary Table 2. [file 41598_2023_28593_MOESM2_ESM.docx]

**Supplementary Table 2.** Sequence of primers used for qRT-PCR.

| Gene symbol | Sequence (5’ – 3’) |
| --- | --- |
| GMactin | F: GGTGAATTATTTTCGCTCCGGG  R: TCAATTTCTCCGAAGCCAGCA |
| Gm02g222400 | F: GGCAATCCACAGAGACGCA  R: CCTAAGAGCTCGCCTGTTGG |
| Gm18g009700 | F: CATATTTGACGCCAAGGCCG  R: CGAAAGGAAAAACCTATAAGGCCG |
| Gm13g361900 | F: TTGGCACCATCACCGCAATA  R: CTTGGGTGCCGAGATCAGAG |
| Gm14g127800 | F: TATTGGATCTGCCCTGTGGC  R: CACCCGTCACCACTAAGCAT |
| Gm07g153100 | F: ATAATCACGACACAAGCAGCAAC  R: GAGTGAACCTATCCTGGTGTCC |
| Gm13g231700 | F: ACCACACCATCGGTTTACCC  R: GTTTTGGCCCACCCACAATC |
| Gm15g011900 | F: GGTGTGCTGTTTGGTTTCGT  R: GGGCAAAGCTCTACTTCCCA |
| Gm15g012000 | F: TGATGACAGTGGGACTGACG  R: TATCCGGTTGGGGAAAGAGTC |
| Gm01g118000 | F: CTCAGGCGGTTCCTGAGAAG  R: GTTTGAAATAGGGAGAAACTCCAGC |
